# HIV RGB: Automated Single-Cell Analysis of HIV-1 Rev-Dependent RNA Nuclear Export and Translation Using Image Processing in KNIME

**DOI:** 10.3390/v14050903

**Published:** 2022-04-26

**Authors:** Edward L. Evans, Ginger M. Pocock, Gabriel Einsdorf, Ryan T. Behrens, Ellen T. A. Dobson, Marcel Wiedenmann, Christian Birkhold, Paul Ahlquist, Kevin W. Eliceiri, Nathan M. Sherer

**Affiliations:** 1 McArdle Laboratory for Cancer Research (Department of Oncology), Institute for Molecular Virology, and Carbone Cancer Center, University of Wisconsin-Madison, Madison, WI 53706, USA; elevans2@wisc.edu (E.L.E.III); pocockgm@gmail.com (G.M.P.); rtbehrens@wisc.edu (R.T.B.); 2 Laboratory for Optical and Computational Instrumentation, Center for Quantitative Cell Imaging, University of Wisconsin-Madison, Madison, WI 53706, USA; mail@gabriel-einsdorf.de (G.E.); ellen.dobson@wisc.edu (E.T.A.D.); marcel.wiedenmann@knime.com (M.W.); 3 Morgridge Institute for Research, Madison, WI 53715, USA; 4 KNIME GmbH, 78467 Konstanz, Germany; christian.birkhold@knime.com; 5 John and Jeanne Rowe Center for Research in Virology, Madison, WI 53715, USA

**Keywords:** human immunodeficiency virus type 1, retrovirus, Rev, Rev response element, Gag, unspliced RNA, RNA regulation, RNA trafficking, RNA nuclear export, translation, XPO1, CRM1, Exportin-1, chromosomal region maintenance-1, live cell imaging, Nuclear Ring Segmentation Analysis and Tracking (NR-SAT), Konstanz Information Miner, KNIME, ImageJ, open-source, image analysis

## Abstract

Single-cell imaging has emerged as a powerful means to study viral replication dynamics and identify sites of virus–host interactions. Multivariate aspects of viral replication cycles yield challenges inherent to handling large, complex imaging datasets. Herein, we describe the design and implementation of an automated, imaging-based strategy, “Human Immunodeficiency Virus Red-Green-Blue” (HIV RGB), for deriving comprehensive single-cell measurements of HIV-1 unspliced (US) RNA nuclear export, translation, and bulk changes to viral RNA and protein (HIV-1 Rev and Gag) subcellular distribution over time. Differentially tagged fluorescent viral RNA and protein species are recorded using multicolor long-term (>24 h) time-lapse video microscopy, followed by image processing using a new open-source computational imaging workflow dubbed “Nuclear Ring Segmentation Analysis and Tracking” (NR-SAT) based on ImageJ plugins that have been integrated into the Konstanz Information Miner (KNIME) analytics platform. We describe a typical HIV RGB experimental setup, detail the image acquisition and NR-SAT workflow accompanied by a step-by-step tutorial, and demonstrate a use case wherein we test the effects of perturbing subcellular localization of the Rev protein, which is essential for viral US RNA nuclear export, on the kinetics of HIV-1 late-stage gene regulation. Collectively, HIV RGB represents a powerful platform for single-cell studies of HIV-1 post-transcriptional RNA regulation. Moreover, we discuss how similar NR-SAT-based design principles and open-source tools might be readily adapted to study a broad range of dynamic viral or cellular processes.

## 1. Introduction

Viruses are obligate intracellular parasites that hijack host cell machineries to express genes, replicate genomes, and generate infectious virions. Understanding virus replication dynamics and identifying sites of virus–host interactions are needed to inform the design and optimization of antiviral strategies. To this end, video microscopy of viral replication in single cells has emerged as a particularly powerful approach because it allows for direct interrogation of specific replication stages as they occur.

HIV-1 is a reverse-transcribing virus (family *retroviridae*, genus *lentiviridae*) and the etiological agent of the acquired immunodeficiency syndrome (AIDS) that results in the deaths of >600,000 people annually [1]. During early infection, HIV-1 converts two copies of an ~9 kb viral RNA (vRNA) genome into a double-stranded DNA genome intermediate that is subsequently integrated into and stored within the host chromatin [2]. HIV-1’s productive phases are subsequently initiated after transcription of a full-length ~9 kb 5′ capped, 3′ polyadenylated viral pre-mRNA that is either subjected to complex alternative splicing to generate the full repertoire of viral mRNAs, or exported from the nucleus, introns-intact, by the viral Rev protein (reviewed in [3,4,5,6,7,8,9,10]). Once in the cytoplasm, unspliced (US) vRNA plays a role as either (1) mRNA translated to synthesize the Gag and Gag-Pol polyproteins that co-assemble to form viral capsids at the plasma membrane, or (2) US vRNA genomes packaged by Gag/Gag-Pol into capsids during the assembly process [11,12,13,14,15].

Single-cell video microscopy has been used to study several independent features or stages of the HIV-1 viral life cycle, including, but not limited to: viral entry [16,17], post-entry events [18,19,20,21,22], transcription [23,24], US vRNA nuclear export [25,26], translation [27,28], vRNA and Gag transport in the cytoplasm [29,30,31,32,33], virus particle assembly [34,35,36,37,38,39,40], and the cell-to-cell transfer of virions [41,42,43,44,45]. To date, however, many of these studies have been focused on single viral activities that occur over relatively short time intervals (e.g., seconds to minutes). Our recent efforts to achieve a more comprehensive, systems-level understanding of HIV-1 replication [25,26,46] have required measuring myriad concurrent and sequential events that take place over hours to days, yielding challenges inherent to handling and analyzing large (gigabyte to terabyte) multichannel imaging datasets.

In this study, big data challenges inherent to achieving a more comprehensive imaging-based understanding of HIV-1’s complex post-transcriptional RNA regulatory stages prompted us to design an unbiased, multicolor imaging-based strategy: HIV “Red-Green-Blue” (HIV RGB), capable of detecting and measuring multiple stages of the HIV life cycle simultaneously, including, but not limited to, US vRNA nuclear export, US vRNA translation in the cytoplasm, and US vRNA, Rev, and Gag transport behaviors in the cytoplasm, prior to and during the process of virus particle assembly. HIV RGB relies upon imaging tagged HIV-1 RNA and proteins using long-term (>24 h) video microscopy prior to image processing, using a new analysis workflow that we call “Nuclear Ring Segmentation Analysis and Tracking” (NR-SAT). NR-SAT represents an intuitive open-source method for single-cell tracking, reliable nucleus vs. cytoplasm cell segmentation, and fluorescence intensity measurements using ImageJ plugins [47,48] integrated into the open source KNIME analytics platform [49]. We describe an experimental HIV RGB setup for visualizing HIV-1’s post-transcriptional stages, provide instructions for extracting single-cell measurements using NR-SAT, and validate the system using a panel of mutated Rev trafficking variants that are hypothesized to perturb the efficiency of viral US vRNA nuclear export.

Useful single-cell applications for HIV RGB include, but are not limited to, viral genetics studies, investigating roles for HIV-1 host regulatory factors, and screening for the effects of antiviral drugs. Moreover, NR-SAT and related design principles should be readily adaptable to study a myriad of other complex and dynamic viral and cellular processes.

## 2. Materials and Methods

### 2.1. Cell Manipulation and Live Cell Imaging

HeLa.MS2-YFP cells were described previously [25] and maintained in Dulbecco’s modified Eagle medium (DMEM), supplemented with 10% fetal bovine serum, 1% L-glutamine, and 1% penicillin/streptomycin. In a typical experiment, 9 × 10^4^ HeLa.MS2-YFP cells were plated per well in eight-well no. 1.5 glass bottom slides (IBIDI, cat #: 80826) one day prior to transfection and imaging. Cells were transfected using polyethylenimine (PEI) (Polysciences, Warrington, PA, USA), 1 mg/mL stock concentration), with transfection mixes consisting of 50 ng pRev-mCherry variant plasmids mixed with 150 ng of pHIV-Gag-CFP.24xMSL. For each 8-sample experiment, 8.8 μL PEI was diluted in 176 μL Opti-MEM (ThermoFisher Scientific, Waltham, MA, USA) and incubated for 5 min at room temperature. Then, 20 μL of this mastermix was added dropwise to each DNA sample to achieve a final ratio of 0.6 μL PEI per 200 ng DNA (i.e., 3:1 volume to mass ratio), mixed, and incubated at room temperature for 20 min prior to adding to cells.

After transfection, cells were incubated at 37 °C for 4 h prior to the addition of Hoeschst 33342 NucBlue Live Cell ReadyProbes (Life Technologies, Carlsbad, CA, USA) to stain DNA (according to manufactures’ instructions) and then incubated for 30 min prior to imaging. Long-term imaging was carried out on a Nikon Eclipse Ti inverted wide-field epifluorescent microscope (Nikon Corporation, Tokyo, Japan), with cells maintained at 37 °C, ~50% humidity, and 5% CO_2_ in a Pathology Devices LIVECELL stage top incubator (Pathology Devices, Inc., San Diego, CA, USA). Images were acquired using an Orca-Flash 4.0 C11440 CMOS camera (Hamamatsu Photonics, Hamamatsu, Japan) and collected using a 20× Nikon CFI Super Fluor (N.A. 0.75) air objective. For high-resolution imaging, 40× Nikon Plan Fluor (NA 1.3) or 60× Nikon Plan Apo (NA 1.4) oil immersion objective lenses were used. Four-color imaging was carried out with the following excitation/emission filter sets (wavelengths in nm): 325–375/435–485 (Hoeschst), 418–442/458–482 (CFP), 490–520/520–550 (YFP), and 565–590/590–650 (mCherry). For quantitative imaging, images were collected at three positions per well, with points selected where cells were at a density of ~40% confluency to account for cell doubling over the 30-h time course.

### 2.2. Image Processing and Computing Resources

ImageJ and KNIME can be downloaded at [50,51], respectively. In this study, ImageJ Bio-Formats [52] and SCIFIO [53] were used to open and read image data in ‘TIF’ and Nikon’s ‘ND2’ formats. Image hyperstacks were ordered so that the first channel contained the nuclear stain/marker, with the remaining channels in the order desired. We automated the channel re-ordering process with ImageJ batch macro scripts, typically by reading ND2 files, re-ordering the channels, and outputting a TIF file.

The HIV RGB KNIME workflow, as described herein, typically requires ~3 GB of system RAM per GB of input data. Processing a large hyperstack within the workflow may exceed the system’s resources, resulting in a halt error. In these instances, we advise removing un-used channels or cropping the time-course images to a smaller size or frame number. To mitigate system resource limits, KNIME can also be configured to utilize as much system memory as possible, thereby achieving the shortest processing times. This goal can be accomplished by editing the “knime.ini” file “Xmx#g” line (where “#” is a number, and “g” denotes gigabytes), changing the number to reflect how many gigabytes you wish to dedicate to KNIME. If feasible, we recommend allocating 3/4 of the total system memory—we typically set “Xmx24g” to allocate 24 GBs out of a 32 GB system.

### 2.3. Nuclear Segmentation and Subcellular Tracking Using NR-SAT

A tutorial explaining the NR-SAT workflow is included as Supplementary Materials Data File S1. The HIV RGB workflow is included in Supplementary Materials Data File S2, with sample data in Supplementary Materials Data File S3 (all files also available at [54]).

## 3. Results

### 3.1. HIV-RGB: A System for Multicolor Visualization of HIV-1’s RNA Post-Transcriptional Regulatory Stages

**Figure 1:**
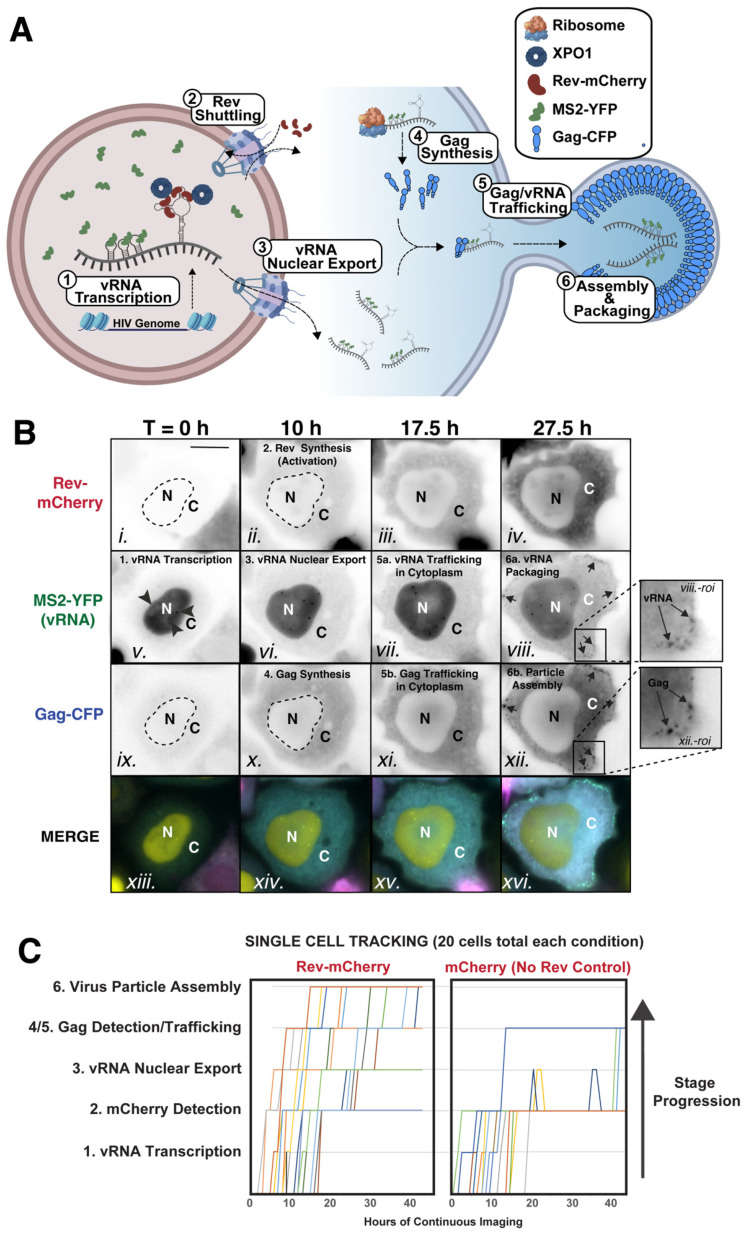
HIV RGB: multicolor single-cell tracking of HIV-1’s post-transcriptional stages. (**A**) Scheme of HIV RGB strategy, wherein nascent vRNA transcripts are visualized in the nucleus using MS2-YFP tagging (Stage 1) prior to Rev-mCherry synthesis (Stage 2) triggering US vRNA nuclear export (Stage 3) and translation of Gag-CFP (Stage 4). Gag and US vRNA then accumulate in the cytoplasm (Stage 5) prior to Gag molecules ultimately multimerizing to form virus particles, packing two dimerized US VRNA genomes (ψ) (Stage 6). Note that stages 2–4 are tightly coupled and proceed almost concurrently. (**B**) Images from live cell imaging of a single HeLa.MS2-YFP cell, demonstrating the progression of stages defined for (A). Here, T = 0 was defined as the acquisition time point just prior to the first detection of Rev-mCherry. Nuclear puncta representative of transcription sites are indicated by black arrowheads in panel v. Black and white images on the right (panels viii. and xii.) demonstrate that vRNA MS2-YFP signals co-localize with Gag-CFP puncta (black arrows), indicating vRNA genome packaging. “N” designates the nuclear compartment and “C” the cytoplasm. Abbreviation “roi” stands for region of interest. Figure 1B corresponds to Video S1. (**C**) Manual tracking of 20 transfected cells, chosen at random, co-expressing Gag-CFP US VRNA with either Rev-mCherry (left) or an mCherry (No Rev) control (right). Each colored line represents a single cell as it progresses to a distinct post-transcriptional stage. Figure 1C corresponds to Video S2.

HIV-1 virion production is initiated after nuclear export of full-length HIV-1 US vRNAs that function in the cytoplasm, either as mRNAs encoding Gag and Gag-Pol proteins that drive assembly of virions or as RNA genomes packaged by Gag and Gag-Pol into assembling virus particles [11,12,13,14,15] (productive phases summarized in Figure 1A). To deliver US vRNAs to the cytoplasm, HIV-1 must overcome strong cellular blocks to the nuclear export of RNA molecules that retain introns [8,55,56,57] and is adapted to do so through the activity of the viral Rev protein—a nucleocytoplasmic shuttling protein that multimerizes on a cis-acting vRNA structure known as the Rev response element (RRE) found in US vRNA transcripts and a subset of viral mRNAs that are partially spliced (PS), encoding the viral envelope, Vif, Vpr, and Vpu proteins [58,59,60]. Rev is a viral early protein synthesized from a fully spliced viral mRNA and targeted to the nucleus through the activity of its N-terminal nuclear localization signal (NLS) [61,62,63,64,65]. Once in the nucleus, Rev multimerizes on the RRE RNA scaffold and recruits Exportin-1 (XPO1, also known as CRM1) through the activity of its C-terminal nuclear export signal (NES), thus activating export of US and PS vRNAs [25,26,66,67,68,69].

Rev is essential to US vRNA nuclear export, operating in trans through its interactions with the RRE. Therefore, we exploited trans-mediated expression of a functional Rev-mCherry fusion protein as a trigger for activation of HIV-1’s late mRNA post-transcriptional stages in the context of our HIV RGB live cell imaging strategy (cartoon depiction in Figure 1A). HIV RGB requires the following core components: (1) an adherent cell line modified to constitutively express low levels of nuclear-targeted MS2 bacteriophage coat protein fused to yellow fluorescent protein (MS2-YFP); (2) a pHIV.Gag-CFP-24xMSL-RRE plasmid expressing HIV-1 US vRNA engineered to express Gag-CFP, bearing 24 copies of the MS2 RNA stem loop (24xMSL), and encoding the Rev Response Element (RRE), but lacking Rev [25,26]; (3) the pRev-mCherry plasmid expressing functional, mCherry-tagged Rev [25,26,69]; and (4) a nuclear stain/marker for single-cell tracking (e.g., Hoechst 33342 live cell stain). We used HeLa cells for this approach (HeLa. MS2-YFP cells) [25,70], because they support efficient HIV-1 replication and have excellent characteristics for long-term live cell imaging—i.e., are stationary, flat, and exhibit large, well-defined nuclear and cytoplasmic compartments amenable to computational cell tracking and segmentation.

Phenotypic read-outs from the HIV RGB system are summarized in Figure 1 (see also Video S1) as follows: First, US vRNA transcription sites are detectable at early time points in the nucleus, indicated by bright puncta that are consistent with of MS2-YFP proteins bound to nascent US vRNAs bearing the 24xMSL cassette (Stage 1, Figure 1A,B(v.) with time of detection in Figure 1C). Rev-mCherry synthesis (Stage 2, Figure 1A,B(ii.),C) then activates US vRNA nuclear export, characterized by accumulation of the MS2-YFP signal in the cytoplasm over time (Stage 3, Figure 1A,B(vi.),C). Concurrent with US vRNA building up in the cytoplasm, Gag-CFP is synthesized (Stage 4, Figure 1A,B(x.),C) and then along with US vRNA, fills the cytoplasm (Stages 5a and 5b, Figure 1A,B(vii.,xi.),C) prior to both signals ultimately accumulating at the plasma membrane in the form of bright puncta consistent with virus particles (Stage 6, Figure 1A,B(viii.,xii.),C).

**Figure 2:**
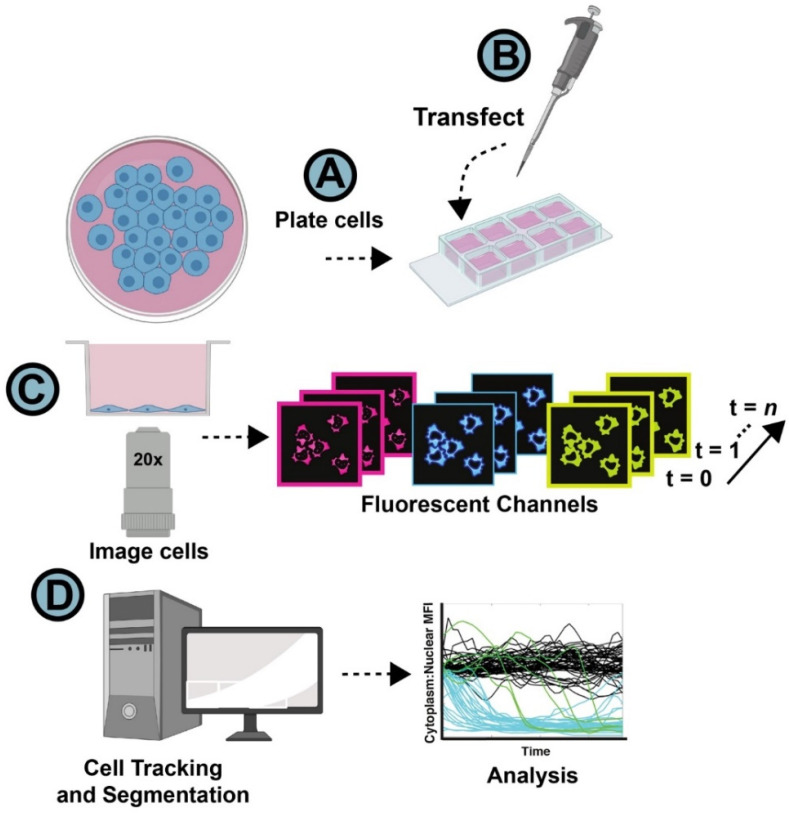
General image acquisition and analysis workflow. (**A**) Adherent cells were plated in 8-well glass-bottomed dishes and allowed to settle for 24 h. (**B**) Cells were transfected to express visible US vRNAs and Rev-mCherry. (**C**) Cells were imaged using multipoint acquisition (3 points per well) 4–6 h post-transfection using a 20× objective, capturing > 100 transfected cells per condition. Images were acquired for each channel over a prescribed time course. (**D**) Images were post-processed for NR-SAT and analysis using KNIME, with data output to graphing software.

To track HIV-1 US vRNA over multiple experimental conditions (e.g., see Rev-mCherry compared to a “No Rev” mCherry control in Video S2), HeLa.MS2-YFP cells were plated at low confluency (30–40%) in 8-well glass-bottomed dishes (Figure 2, step 1) and transfected to express Gag-CFP.24xMSL-RRE US vRNAs with or without Rev-mCherry 4–6 h prior to live cell imaging (Figure 2, step 2). At this point in time, cells were labeled for tracking with Hoeschst 33342 live cell DNA stain. We typically acquired at least three fields for each of the 8 wells in four channels (mCherry/596 nm excitation, YFP/514 nm, CFP/458 nm, Hoechst/405 nm) per sample using a 20× objective lens, allowing for acquisition of >100 expressing cells per condition, per experiment—assuming a transfection efficiency of ~50% (Figure 2, step 3). Images were recorded for >24 h at regular time intervals prior to post-processing, cell tracking, and image analysis (Figure 2, step 4). More details regarding the cell preparation and imaging procedures are given in the materials and methods section. Typical time frames spanned anywhere from 30 min to every 2 h, depending on the temporal dynamics of the planned experiment.

To summarize, the HIV RGB system allows for reliable detection and comparative studies of Rev trafficking, Rev-driven US vRNA nuclear export, US vRNA translation, and Gag synthesis during, and prior to, virus particle assembly. We noted CFP expression in ~10% of HeLa.MS2-YFP cells expressing US vRNA, even in the absence of Rev-mCherry (See “No Rev Control-mCherry Alone” in Figure 1C, and Video S2). Interestingly, however, we did not observe Gag-CFP+ virus particle assembly in these cells, suggesting that these instances most likely reflected rare instances of aberrant US vRNA splicing.

### 3.2. Automated Analysis of HIV-1 RNA Regulation Using NR-SAT and KNIME: An Overview

Subcellular feature segmentation and cell tracking have long been a challenge as they can generate cumbersome quantities of imaging data that often require expensive, proprietary software for analysis. To configure an unbiased, freely available and accessible automated method to allow for rapid processing of HIV-1 gene expression and subcellular trafficking, we chose to build our image processing pipeline using ImageJ image processing functions supported via the KNIME Analytics Platform [49]. KNIME provides a visual editor wherein functions or operations (referred to as ‘nodes’) are connected to create a visual representation of a workflow. Within KNIME, ImageJ functions can be interconnected along with KNIME’s built-in data analysis tools to automate a variety of image processing and segmentation workflows. Both ImageJ and KNIME are open-source tools, freely available to download at [50,51], respectively.

The KNIME Image Processing extension integrates ImageJ image pre-processing, subcellular segmentation, cell tracking, and extraction of cellular morphological feature descriptors for single-cell analysis [49]. The goal of HIV RGB is to segment multi-channel images from viral videos and derive paired mean fluorescence intensity (MFI) measurements for each viral marker (Rev-mCherry, MS2-YFP or Gag-CFP) in the nuclear or cytoplasmic cellular compartments over time and across all channels. To this end, our KNIME NR-SAT workflow was configured to accept multi-channel data with the only requirement being that channel 1 (nuclear channel) contained a nuclear marker/stain for masking. The subsequent channels (measurement channels) can be arranged in any given order. Detailed instructions for NR-SAT are featured in the accompanying HIV RGB/NR-SAT Tutorial (Supplementary Materials Data File S1). 

To test drive the workflow, after installing KNIME, download the HIV RGB/NR-SAT workflow (Supplementary Materials Data File S2, or at [71]) and our test sample data (Supplementary Materials Data File S3). Open KNIME and import the workflow at File > Import KNIME Workflow. Upon opening the workflow, a dialog box will request permission to download additional software dependencies required to run the workflow. You will be asked to locate and install the required ImageJ Integration package, with details provided in the tutorial (Supplementary Materials Data File S1).

**Figure 3:**
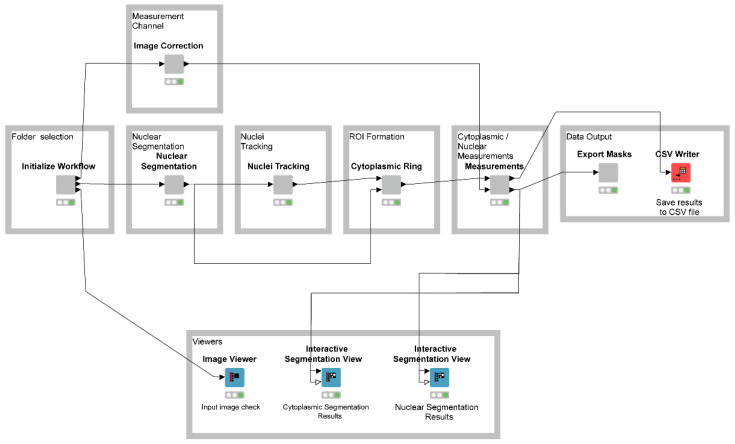
HIV RGB node and component overview. The HIV RGB workflow is organized into several sections and components. The “Initialize Workflow” section loads the image stacks to be processed. These data are then passed to the “Measurement Channel” and “Nuclear Segmentation” components, respectively. The “Measurement Channel” component processes all measurement channels (default: background subtraction), while the “Nuclear Segmentation” component only processes the nuclear channel. The nuclear channel is first segmented, tracked, and then passed to the “ROI Formation” channel where the cytoplasmic rings are generated. Finally, the data derived from the “Measurement Channel” is rejoined to the now-generated nuclear masks and cytoplasmic rings, and measurements are made followed by data output in a CSV formatted file.

The HIV RGB/NR-SAT workflow consists of six major sections: (1) loading data, (2) nuclear segmentation, (3) selecting a measurement channel, (4) nuclei tracking, (5) ROI formation, and (6) perinuclear/nuclear measurements (Figure 3). These sections are organized into components, which in themselves contain a varying number of internal nodes that can be independently configured. All components within the workflow are annotated with a brief description of each node’s function. By default, the workflow accepts multi-channel live cell microscopy data, acquired at any magnification (our test dataset was acquired using a 20×, N.A. 0.75), where channel 1 contains the nuclear marker/stain and channel(s) *n* contain the signal of interest. Prior to executing the workflow, we recommend that user-generated live cell data be re-ordered to fit these parameters. Alternatively, there are locations within the workflow wherein nodes can be modified for channels to best fit the incoming data, as described in the tutorial (Supplementary Materials Data File S1).

**Figure 4:**
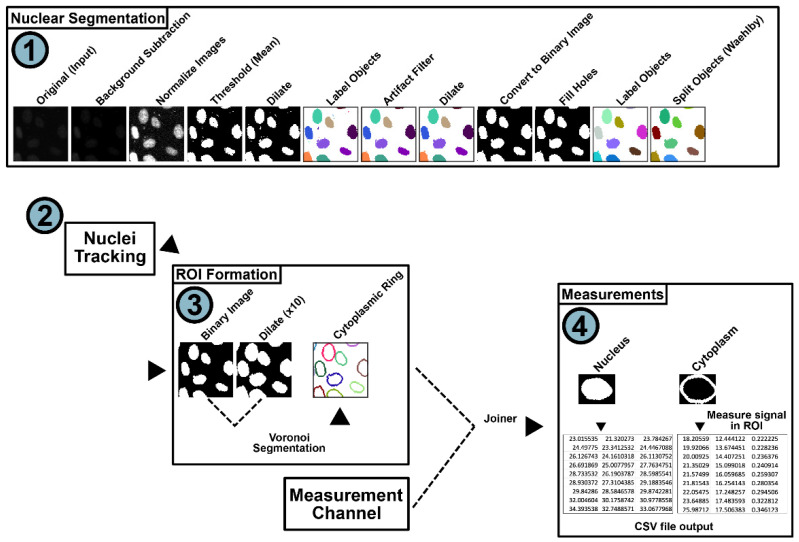
NR-SAT single-cell segmentation and tracking scheme. (Phase 1) The input nuclear channel is illumination-corrected using background subtraction, followed by local contrast enhancement. The resulting images then undergo thresholding (Mean method) and are dilated and then labeled. An object filter is applied to remove threshold artifacts prior to converting the images into binary images and filling holes. Finally, the data is passed to the Wählby Cell Clump Splitter, which separates nuclei that are in proximity to one another. (Phase 2) These data are then passed to the nuclei tracking nodes where the ImageJ plugin TrackMate is implemented to track each cell, accounting for splitting and merging events. (Phase 3) After the nuclei are tracked, the nuclear mask is duplicated and dilated (number of dilations is user-defined and applied to all images in the same manner, 10× in this example) to generate two masks, where the newly dilated mask is larger than the original source mask. These two masks (the newly larger dilated mask and the smaller original mask) are then segmented via the Voronoi segmentation node, generating cytoplasmic rings that are approximately a fixed pixel-width. (Phase 4) Finally these cytoplasmic rings, along with the nuclear masks, are applied to the measurement channel(s) and written to a CSV output file.

For HIV RGB cell segmentation using NR-SAT, the nuclear channel was illumination-corrected prior to image intensity normalization/equalization, binary thresholding (means method), object labeling, and object splitting via the Wählby method (Figure 4, phase 1) [72]. If the order of channels did not conform to this recommended configuration, the nuclear marker channel could be selected manually using the “Column Filter” node. After processing the input image data, the “Nuclear Segmentation” component outputs describing unlinked nuclear labels were transferred to the “Nuclei Tracking” component. The labeled image data were then processed using the “TrackMate Tracker” [73]. The labeled objects (e.g., identified nuclei) were then tracked (Figure 4, phase 2). Labels were linked and tracked over time based on user-configurable parameters, such as distance between two objects and the splitting and merging of objects. The outputs of this component were linked and tracked using individual nuclei labels.

After tracking, the nuclei labels were passed on to the cytoplasmic ring component. The cytoplasmic ring component then took the labeled nuclei produced from phase 1, dilated each nucleus (user set value), and joined the mask to the original un-dilated nucleus. These tracked nucleus masks were used to generate perinuclear cytoplasmic rings using the “Voronoi Segmentation” component (Figure 4, phase 3), with each ring’s width based on the number of dilation iterations performed (default: 10) that, in turn, track their respective nuclei. Increasing the number of nuclei dilation iterations increases the thickness/width of the generated cytoplasmic ring. Decreasing the number of nuclei dilation iterations will, conversely, decrease the cytoplasmic ring thickness/width. While this approach does not, unfortunately, measure the entire cytoplasm, its advantage for automated processing of dynamic live cell imaging data is its reduction in erroneous segmentation of two or more adjacent cells. Attempting to segment the entire cytoplasmic signal, using thresholds from individual cells, frequently fails by being unable to differentiate two cells that are in proximity. This issue is especially problematic as the duration of the imaging experiment grows and cells replicate, increasing the likelihood that cells will make contact with each other and ultimately fail to segment properly.

Next, the dilated nuclei along with the original nuclei masks were processed by the ImageJ Voronoi segmentation node [73], which outputs a “cytoplasmic ring” around each nucleus that allows for reliable measurements of perinuclear cytoplasmic MFI (Figure 4, phase 3, see also Video S3). Simultaneously, the measurement channel was processed and joined to the nuclei segmentation and tracking data, creating a table that defines nuclear and cytoplasmic ring masks for each the measurement channels. Finally, nuclear masks and cytoplasmic ring masks were applied to the measurement channels with MFI measurements made and then written out as a standard comma-separated values (CSV) file (Figure 4, phase 4) that can be read using conventional spreadsheet data analysis programs. In the presented workflow, measurement channels underwent background subtraction prior to measuring the signal in the cytoplasmic and nuclear compartments. Additional image processing operations that only impact the measurement channels could be added if relevant to the input data by adding processing nodes in the “Image Correction” component.

### 3.3. Case Study: Altered Steady-State Rev Subcellular Distribution Has Only Minor Impact on HIV-1 US RNA Transport and Translation

**Figure 5:**
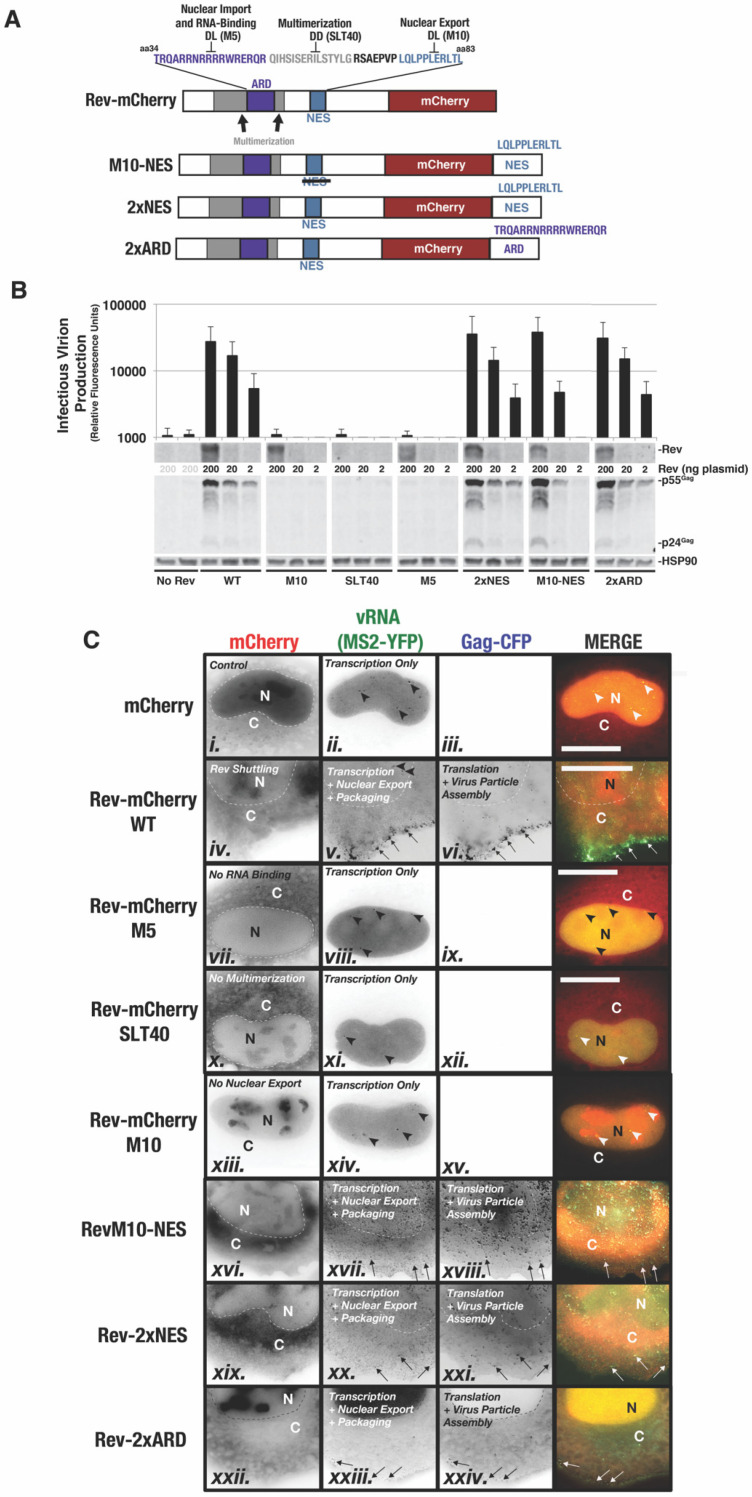
Rev-mCherry functional mutants and trafficking variants used to test the HIV RGB strategy. (**A**) Depiction of Rev-mCherry fusion protein and key functional domains. The arginine-rich domain (ARD) (indicated in purple) encodes residues important for Rev’s nuclear import and binding to the RRE. The ARD is flanked by sequences involved in Rev-Rev multimerization (indicated in gray) to form a functional export complex. Rev’s nuclear export signal (NES) (indicated in blue) binds to XPO1. The Rev M5 mutant bears a R42D/R43L to substitution in the ARD that abrogates Rev-RRE binding. SLT40 encodes I59D and I60D substitutions reducing Rev’s capacity to self-associate. The Rev-M10 mutant bears an L78D/E79L substitution in the NES that abolishes Rev’s ability to interact with XPO1. (**B**) Functional assessment of Rev-mCherry variants used in this study. To gauge infectious virion production, HIV-1 virions were generated using a HEK293T viral infectivity assay wherein we generated Rev-inactivated HIV-1 YFP reporter virus in the presence or absence of *trans*-delivered Rev-mCherry or the indicated Rev-mCherry variant protein. Supernatants were harvested at 48 h post-transfection and used to infect target cells, with infectivity measured based on the YFP reporter. Lysates were also harvested from producer cells at 48 h for immunoblot-based detection of HIV-1 Gag, Rev-mCherry, and HSP90 (loading control). (**C**) Images of individual HeLa cells acquired at 24 h post-transfection and expressing US vRNA in the presence or absence of Rev-mCherry or the indicated Rev trafficking variants. Transcripts are visible in the nucleus for all conditions (see frames ii., v., viii., xi., xiv., with bright nuclear puncta indicated in some images with arrowheads). Only the Rev-mCherry variants deemed functional based on (**B**) were competent to activate US vRNA nuclear export (panels v., xvii., xx. and xxiii.), Gag-CFP synthesis (panels vi., xviii., xxi. and xxiv.), and virus particle assembly (panels vi., xviii., xxi. and xxiv, see thin arrows). “N” designates the nuclear compartment and “C” the cytoplasm. Images represent compressed z-stacks so that some of the images (notably for Rev-M10-NES, Rev-2xNES, and Rev-2xARD) include signals from the ventral plasma membrane.

**Figure 6:**
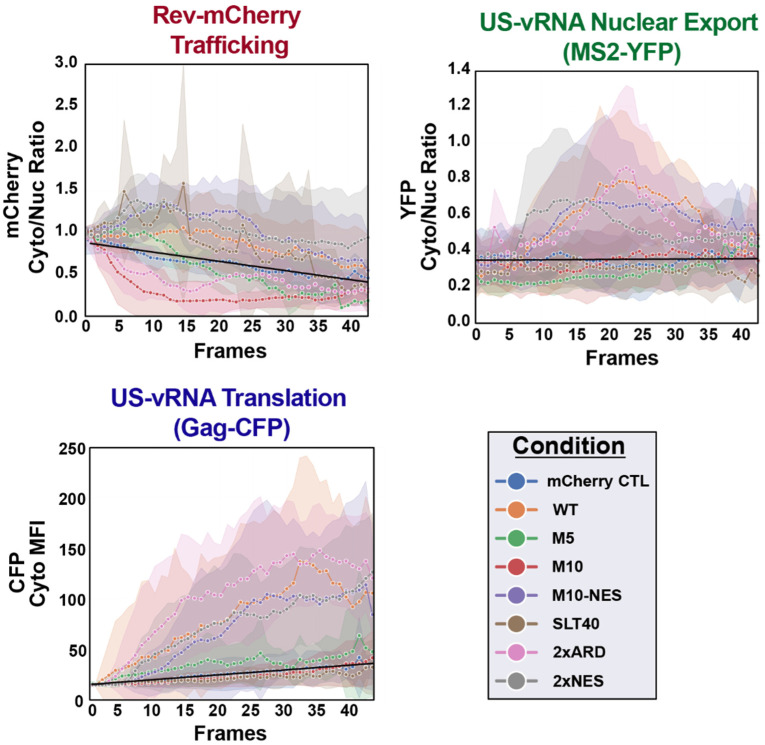
NR-SAT output. Color-coded traces for unbiased HIV RGB analysis of the effects of individual Rev-mCherry variants on Rev localization (top left), US vRNA nuclear export (top right), and US vRNA translation to generate Gag-CFP (bottom left). Cells were imaged for 24 h, with images processed using the NR-SAT workflow, tracking the relative subcellular location of each Rev-mCherry variant or the mCherry control (black line). Lines indicate mean fluorescence intensity (MFI) with standard error of the mean (SEM) for each time point, shown as a similarly colored delimited background for each condition.

To validate HIV RGB using NR-SAT as an unbiased system for analysis of HIV-1 US RNA regulation, we tested the effects of perturbing Rev-mCherry function or steady state subcellular localization on US vRNA nuclear export and translation (Figure 5 and Figure 6). Rev encodes three core functional domains (cartoon depiction in Figure 5A): (1) an arginine-rich domain (ARD, shown in purple) encoding overlapping nuclear/nucleolar localization signals (NLS/NoLS) and Rev’s RNA binding domain; (2) hydrophobic regions that flank the ARD to mediate Rev-Rev multimerization (shown in gray); and (3) the NES, located in the C-terminal half of the protein, that binds XPO1 to form a functional US RNA viral ribonucleoprotein nuclear export complex (shown in blue). We compared the effects of wild-type Rev-mCherry (WT) to an mCherry (“No Rev”) control and the six Rev variants described in Figure 5. Three carried inactivating mutations either within Rev’s ARD (mutant “M5”) [59], multimerization domain (mutant “SLT40”) [74], or NES domain (mutant “M10”) [59]. The other three variants represented Rev “trafficking” mutants, wherein either a second ARD (“2xARD”) was appended to the C-terminus of Rev-mCherry or a second NES (“2xNES”) was appended to the C-terminus of either Rev-mCherry or RevM10-mCherry [26,69].

We first confirmed that the M5, SLT40, and M10 mutations were unable to activate Gag expression and infectious virion production from a Rev-minus HIV-1 reporter virus (Rev trans-complementation assay, Figure 5B), as expected. Interestingly, the three trafficking mutants (M10-NES, 2xARD, and 2xNES) had little net overall effect on Gag or virion infectivity levels compared to WT. However, M10-NES exhibited lower activity than WT at its lowest levels of expression, consistent with prior findings [26] (Figure 5B, compare M10-NES and WT at 2 ng). We also evaluated the distribution of each Rev-mCherry variant relative to MS2-YFP-tagged viral US RNA and Gag-CFP in our HIV RGB configuration in fixed cells, analyzed at 24 h post-transfection (Figure 5C). As anticipated, wild-type Rev-mCherry (WT) exhibited a diffuse cytoplasmic distribution and was also detected in the nucleus enriched at nucleoli—RNA-rich subnuclear organelles that serve as sites of ribosome biogenesis (22) (Figure 5C, panel iv.). The Rev M5 and SLT40 mutants were predominantly cytoplasmic, consistent with defects to RNA binding and/or nuclear import (Figure 5C, panels vii. and x.). Rev-M10 was restricted to the nucleus and nucleoli, consistent with a defect to XPO1 engagement (Figure 5C, panel xiii.). None of these Rev-inactivating mutations triggered US RNA nuclear export or Gag-CFP expression, with phenotypes similar to the Rev-minus (mCherry alone) control (Figure 5C, panels i.–iii.). However, we readily detected vRNA transcription for all three conditions, indicated by the formation of numerous bright MS2-YFP puncta observed in all cells at 24 h post-transfection (Figure 5C, e.g., see arrows in panels ii., viii., xi. and xiv.). Appending the NES to M10 (NES-M10) rescued Rev’s trafficking to the cytoplasm (Figure 5C, panel xvi.); while the addition of the second ARD (2xARD) or NES (2xNES) biased Rev’s distribution to the nucleus or cytoplasm, respectively, as expected (Figure 5C, panels xix. and xxii.). Consistent with the *trans*-complementation infectivity assay (Figure 5B), US vRNA nuclear export, Gag-CFP synthesis, virus particle production, and RNA genome packaging were observed for each of the conditions including a functional Rev variant (Figure 5C, v.–vi., xii.–xiii., xx.–xxi., xxiii.–xiv.).

NR-SAT plots of live single-cell data confirmed detection and reliable tracking of Rev subnuclear localization and US vRNA nuclear export over time in an unbiased manner for each tested variant (Figure 6, with individual plots for each condition in Supplementary Materials Data File S4). As expected, WT, SLT40, M10-NES, and 2xNES Rev-mCherry variants were more cytoplasmic over 30 h of imaging, relative to the mCherry fluid phase control (black line), while M10 and the 2xARD were predominantly nuclear (Figure 6, top left panel). Rev-M10 exhibited a profound nuclear signal bias over time, consistent with its lack of binding to XPO1 (Figure 6, top left panel, red). As expected, 2xARD was also predominantly nuclear over the time course (Figure 6, top left panel, pink).

Plotting US vRNA (MS2-YFP) signals for mCherry-positive cells also generated expected results for WT, 2xARD, 2xNES, and M10-NES, demonstrating a reliable shift in MS2-YFP signals from the nucleus to the cytoplasm between 5–10 h post-infection, which was not observed for the M10, SLT40, or M5 negative controls (Figure 6, top right panel). Congruent with this result, we only observed notable increases to Gag-CFP signals in the cytoplasm when the US vRNA was expressed in the presence of the active Rev variants (WT, 2xARD, 2xNES, and M10-NES) (Figure 6, bottom left panel). These results validated our HIV RGB workflow as capable of detecting Rev, US vRNA, and Gag-CFP simultaneously in several cells, while also defining their subcellular compartmentalization and measuring their relative abundance in each compartment over time. Biasing Rev’s subcellular distribution with the addition of a second ARD or NES did not have a major impact on single-cell US vRNA nuclear export or Gag-CFP dynamics. However, we did note that the 2xARD variant consistently yielded moderately higher levels of Gag-CFP expression.

## 4. Discussion

Herein, we describe an unbiased open-source quantitative imaging strategy for studying HIV-1 US RNA regulation in the context of single cells. Image analysis using ImageJ functions and plugins in the context of a KNIME workflow represents a powerful and user-friendly method to extract dynamic fluorescent protein signatures over time for large and complex multi-channel imaging datasets [49]. ImageJ is already a widely used and functionally sophisticated open-source software package for image processing. However, it is configured for complex, high-throughput applications. Coupling ImageJ with the KNIME Analytics Platform establishes an intuitive, user-friendly interface, wherein ImageJ functions can be visually represented, linked, reconfigured, and scaled as needed for the task at hand.

For the HIV RGB strategy, our initial goal was to establish reliable nuclear and cytoplasmic segmentation to measure HIV-1 post-integration trafficking dynamics. To do so, we developed NR-SAT, using nuclear mask dilation to generate a cytoplasmic ring that allowed us to differentiate proximal nuclear and cytoplasmic regions, thereby obviating the need to track cell edges or the entire cytoplasmic region, which can be highly variable depending on an individual cell’s morphology and cell plating density [75,76]. In the HIV RGB test case presented here (Figure 6), we showed how the approach allowed us to track three independent viral components (Rev, US vRNA, and Gag) simultaneously in many single cells to delineate real-time detection of Rev in either the nucleus or cytoplasm, US vRNA transitions from the nucleus to the cytoplasm, and subsequent US vRNA translation in the cytoplasm to generate Gag proteins. We also confirmed that Rev steady-state localization has little to no effect on US vRNA nuclear export and Gag translation kinetics, reinforcing the plasticity of the Rev/XPO1 system [77,78], which seems to be more sensitive to Rev abundance and shuttling capacity (see Figure 5B) relative to where the Rev is predominantly localized within the cell [26,69].

As noted above, mask dilation during NR-SAT works well for measuring diffuse signals, such as those that predominate in our HIV RGB system, but are less able to measure events that are highly localized (e.g., viral activities distal to the nucleus including virus particle assembly at the plasma membrane). There are also caveats to our transfection-based approach, wherein, although trends observed for Rev variants were consistent with expectations (Figure 6), there was significant per-cell heterogeneity potentially reflecting differences to per-cell plasmid uptake ratios. Therefore, some caution needs to be taken in that per-cell Rev or US vRNA levels may or may not reflect levels achieved during actual infection.

However, because the software is modular, a future goal will be to modify KNIME and ImageJ plugins to detect and monitor viral US vRNAs as they are transcribed at authentic sites of proviral integration into the chromosomes. We are also augmenting the strategy to include novel machine learning and co-localization algorithms, thus providing unbiased approaches to pinpoint key viral transitions in the context of single cells—such as the direct onset of virus particle assembly and sites of dynamic interplay between viral and/or cellular factors (e.g., sites of Gag-US vRNA or Gag-envelope interactions). Moreover, the platform should have a utility for drug target development based on the capacity of multi-stage imaging to identify precise mechanisms of action (e.g., how and where does the drug affect RNA trafficking, stability, or translation?), as well as its tractability for high-content, imaging-based screens in conjunction with small molecule libraries or CRISPR/Cas9-based gene modulation.

We are also confident that variations on this approach can be used to study nuclear export and translation for many other combinations of tagged viral or cellular RNA or proteins, provided that the tags do not exhibit spectral overlap, do not perturb the RNA or protein trafficking, and that the system conforms to the limitations of the NR-SAT ring (i.e., improvements would be needed to track localized transport in association with the cytoskeleton or organelles, etc.). Moreover, the best results would be obtained using cell lines that are (1) large and flat, allowing for precise nucleus/cytoplasm cell segmentation, and (2) largely stationary, allowing for imaging the exact same cells over hours to days. Results from this method would also be dependent on image quality, cell seeding density, illumination, etc.

In summary, we described an open-source analysis system to unify independent measures of multiple relevant RNAs and proteins in single cells and in real time to reveal critical cell and molecular biology aspects of selected pathways, as in the specific example developed here to delineate essential HIV–host interactions and a more complete model of the HIV lifecycle.

## Data Availability

The “HIV RGB with NR-SAT” tutorial, KNIME workflow and test data are available for download at [71].

## Supplementary Materials

The following supporting information can be downloaded at: https://www.mdpi.com/article/10.3390/v14050903/s1, Video S1: Phenotypic read-outs from the HIV RGB system. Video S2: Tracking HIV-1 US vRNA nuclear export and translation in the presence or absence of Rev and mutated Rev variants. Video S3: Example of NR-SAT. Supplementary Data File S1: Tutorial: Using HIV RGB with NR-SAT in KNIME. Supplementary Data File S2: HIV RGB with NR-SAT in KNIME workflow. Supplementary Data File S3: HIV RGB test dataset. Supplementary Data File S4: NR-SAT output data for Rev variants shown in Figure 6.
