# Supplementary material for: HIV RGB: Automated Single-Cell Analysis of HIV-1 Rev-Dependent RNA Nuclear Export and Translation Using Image Processing in KNIME
Source: Viruses. 2022 Apr 26;14(5):903. doi: 10.3390/v14050903 (PMC9145009; doi:10.3390/v14050903)
Supplement: Supplementary file 1 [file viruses-14-00903-s001.zip › Supplementary Data File S1.pdf]

KNIME Image processing tutorial

1. Download and install KNIME

Visit www.knime.com and download KNIME for your platform (Windows, MacOS and Linux are supported).

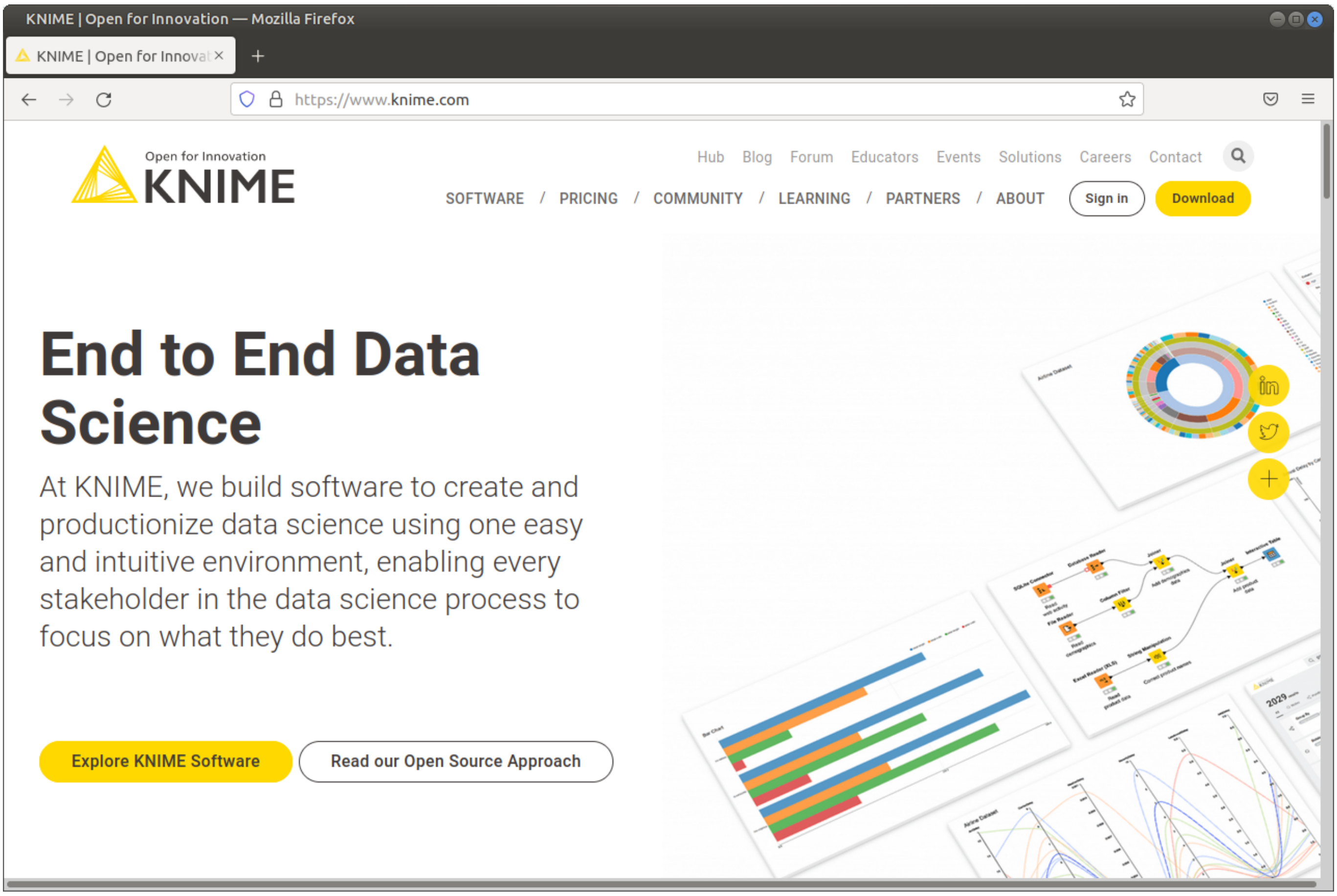

2. Launch KNIME

Launch KNIME and setup up your workspace directory. This directory will store the workflow associated files.

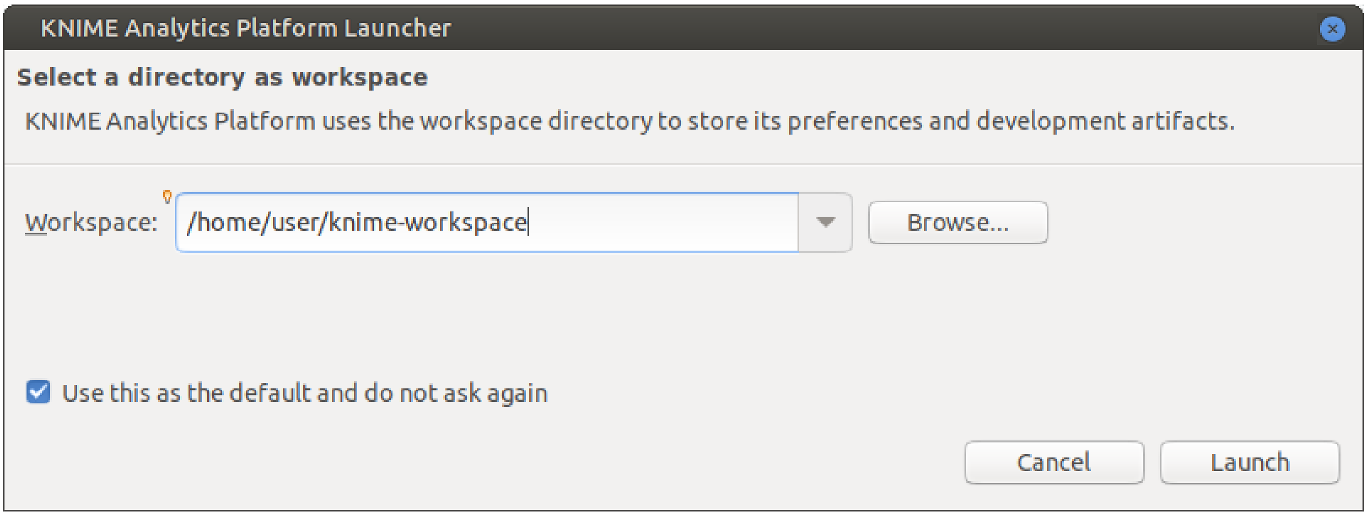

KNIME will open with several side panels along with a welcome screen. How to use each of these panels is beyond the scope of this tutorial. For more information on editing/developing workflows please visit the KNIME development documentation. In this tutorial we will use the `KNIME Explorer` side panel to select and load a workflow.

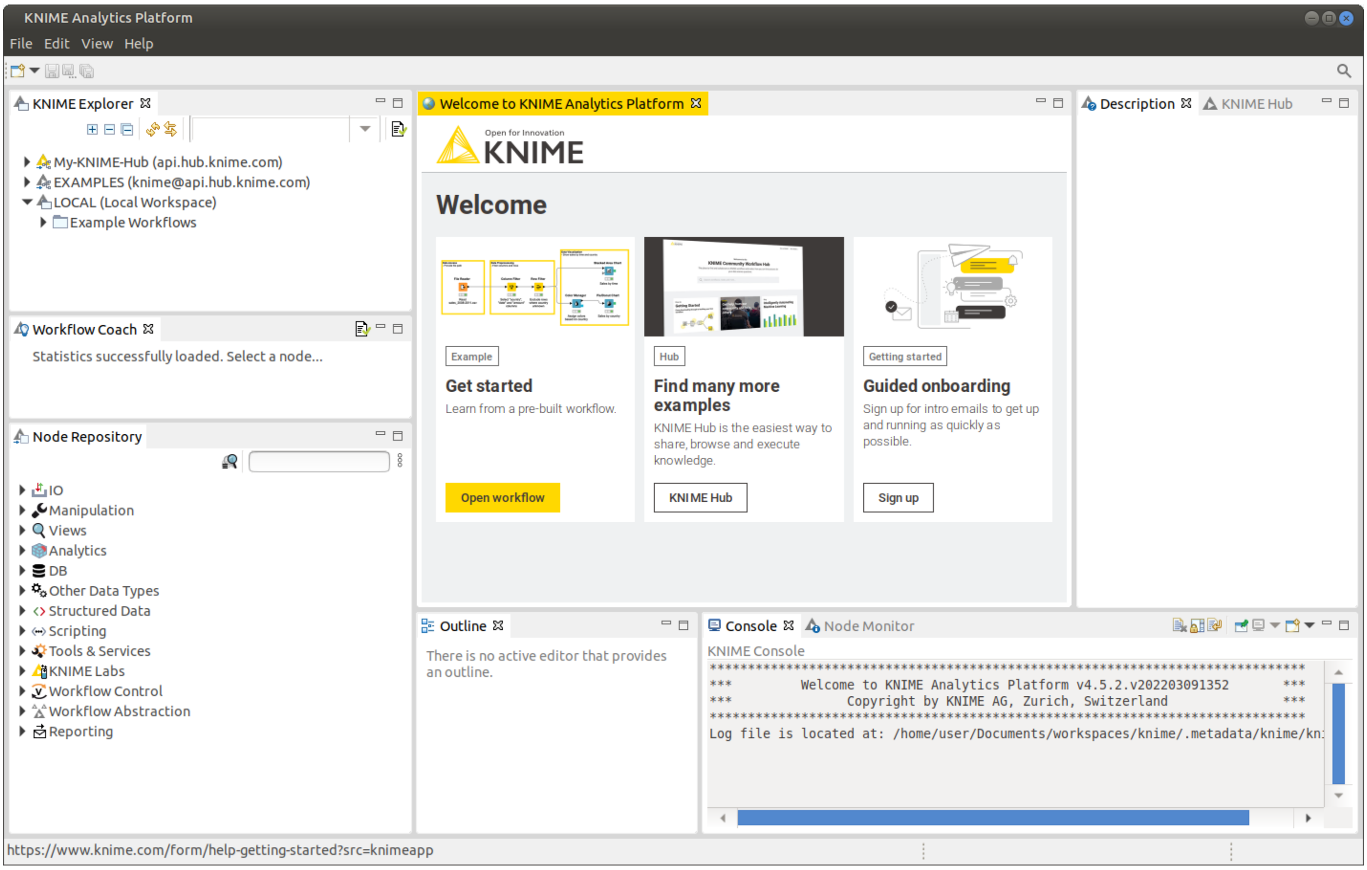

3. Load a workflow

You can load the NR-SAT the workflow by visiting the KNIME hub at hub.knime.com and search for `NR-SAT`. Then using the "Drag Workflow" feature to drag the workflow into the `KNIME Explorer` side panel. Alternatively you can access the NR-SAT from the HIV RGB GitHub repository at github.com/shererlab/hivrgb. The NR-SAT workflow and sample data (Supplementary Data File 2 and 3 respectively) are also included with the the HIV RGB publication. The workflow can also be downloaded and directly imported into KNIME (**File** > **Import KNIME Workflow...**).

Once the workflow as been added to the KNIME workspace, double click the workflow to open and load it.

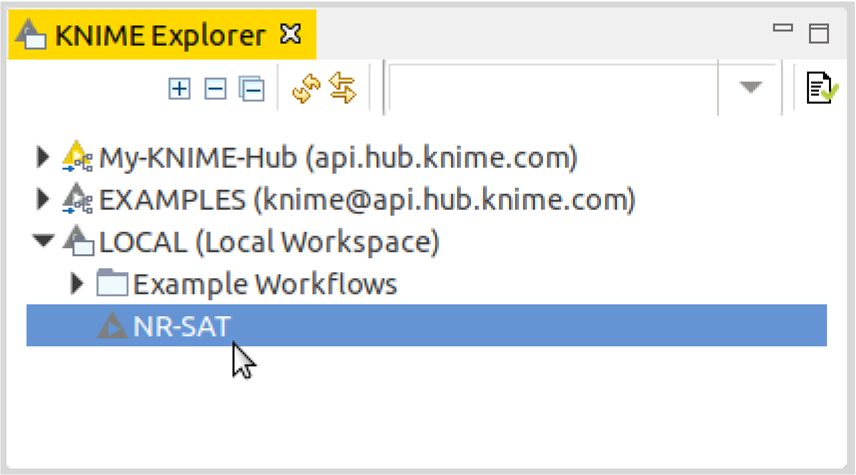

Before you can begin using the NR-SAT workflow, several dependencies need to be resolved. Click `Yes` to automatically install most of the required extensions.

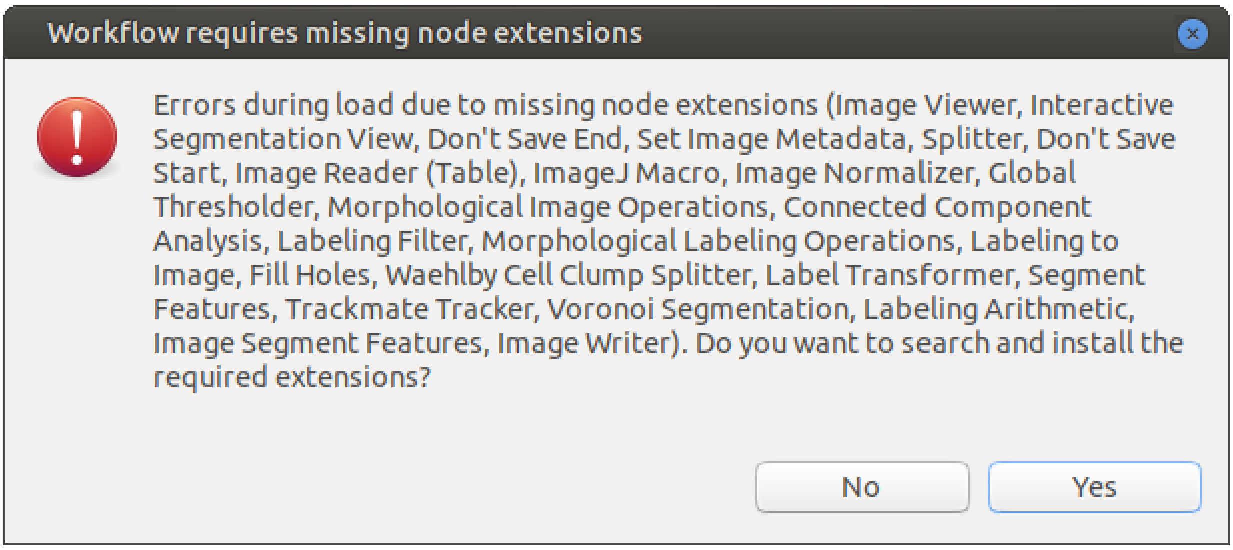

A new window will display which packages are being installed and their associated details.

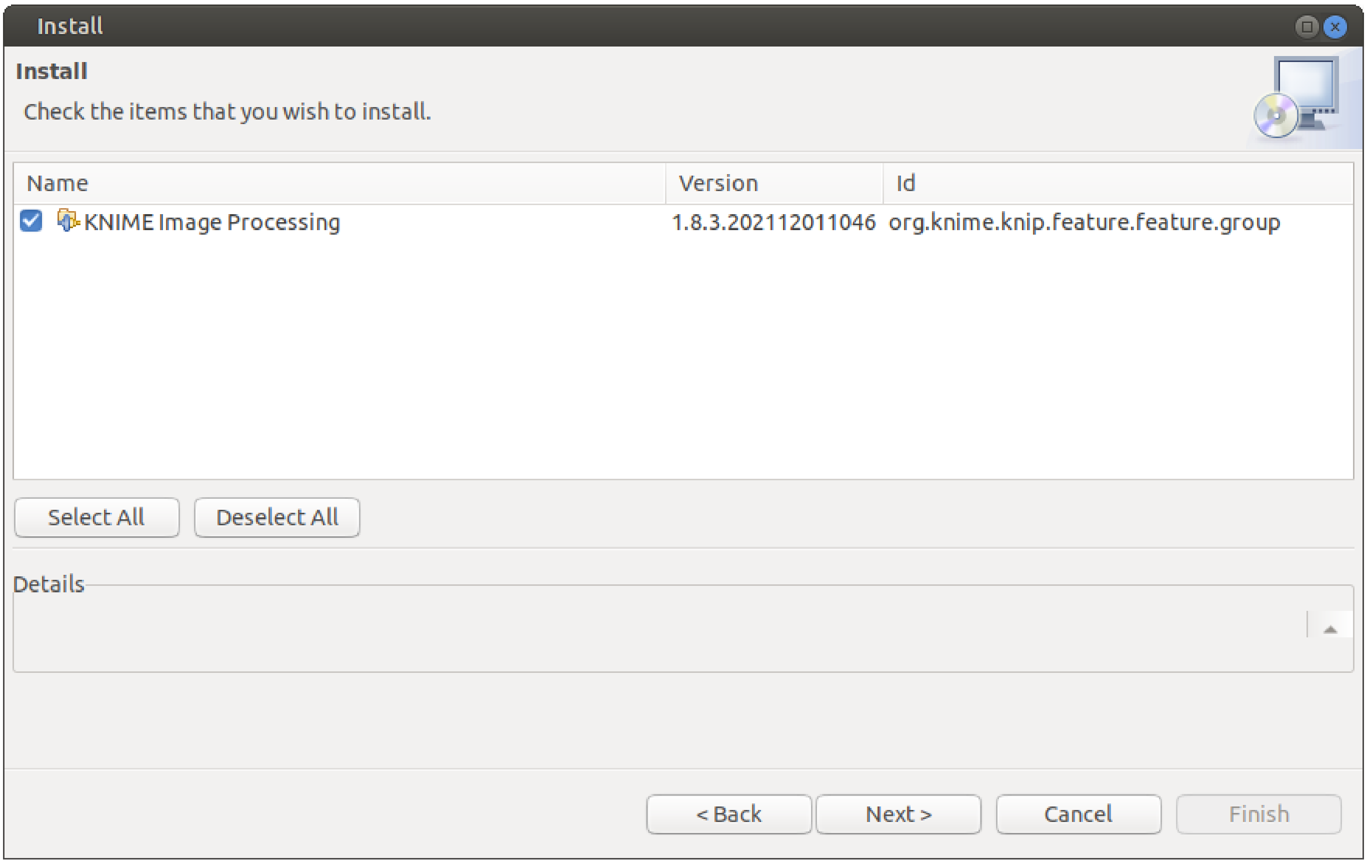

After installing the `KNIME Image Processing` extensions you may receive a "Restart KNIME" notification. Click `Restart Now` and continue the tutorial after KNIME has successfully started again.

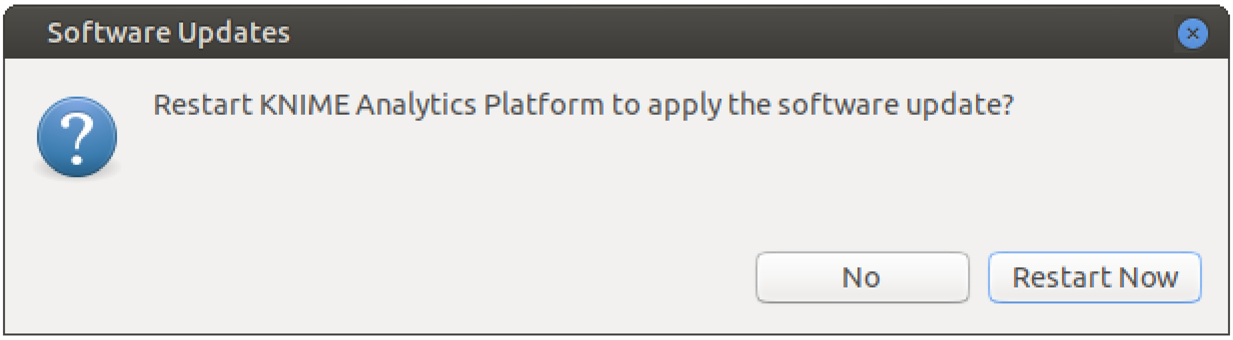


```
Note that you may receive an error indicating that ImageJ Macro was not
found. Click OK and continue with the tutorial. The ImageJ integration
steps below will resolve this issue.
```

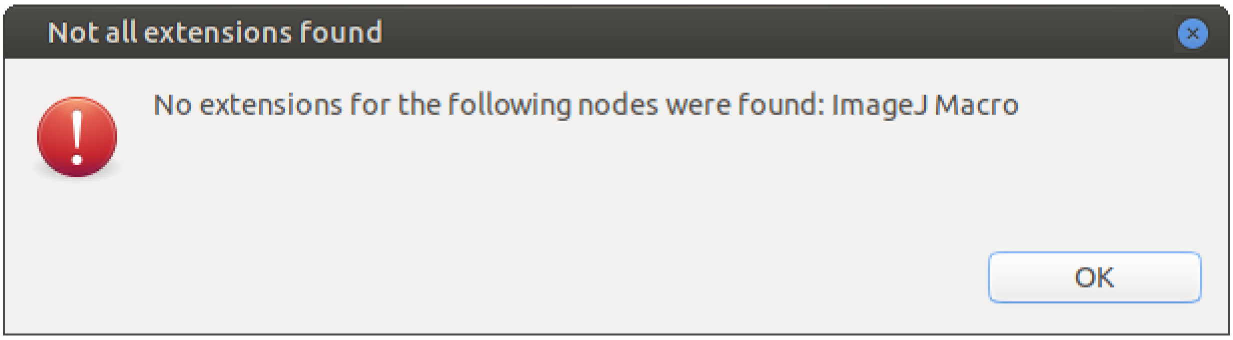

Before you can run the NR-SAT workflow, ImageJ integration must be manually enabled. Click **Help** > **Install New Software...** to open the software management window.

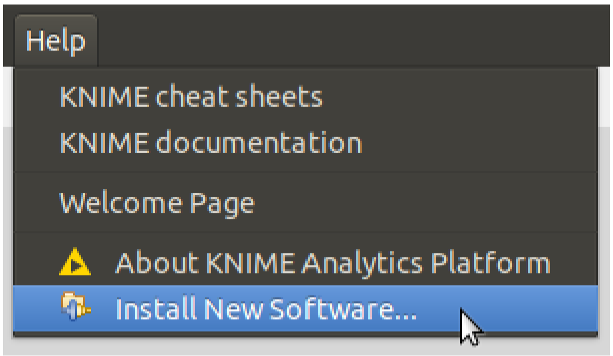

Next click the **Manage...** (red arrow) to open the software sources management window.

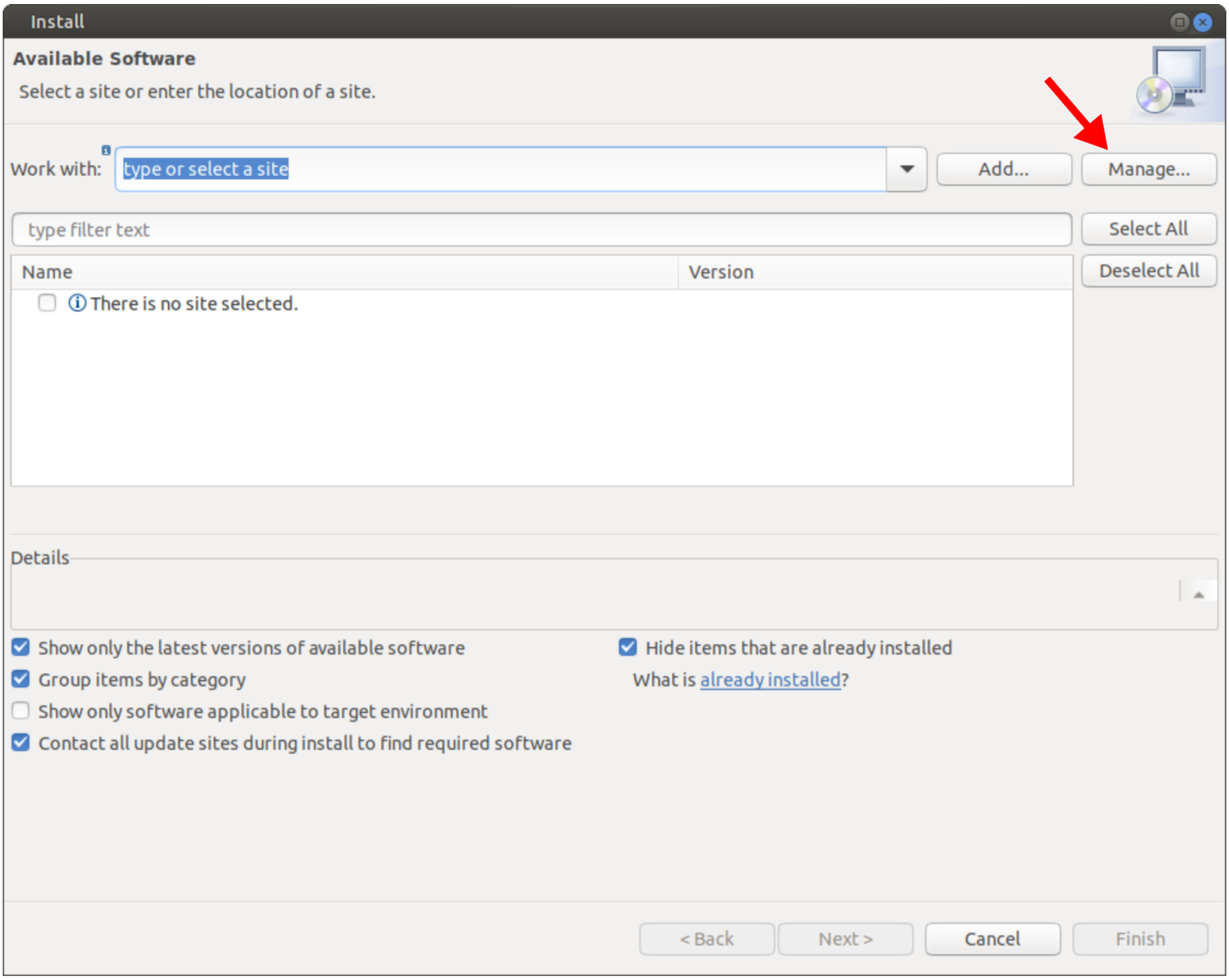

Click the check box next to the `KNIME Community Extensions (Experimental)` software site (red arrow). To accept this settings click **Apply and Close**.

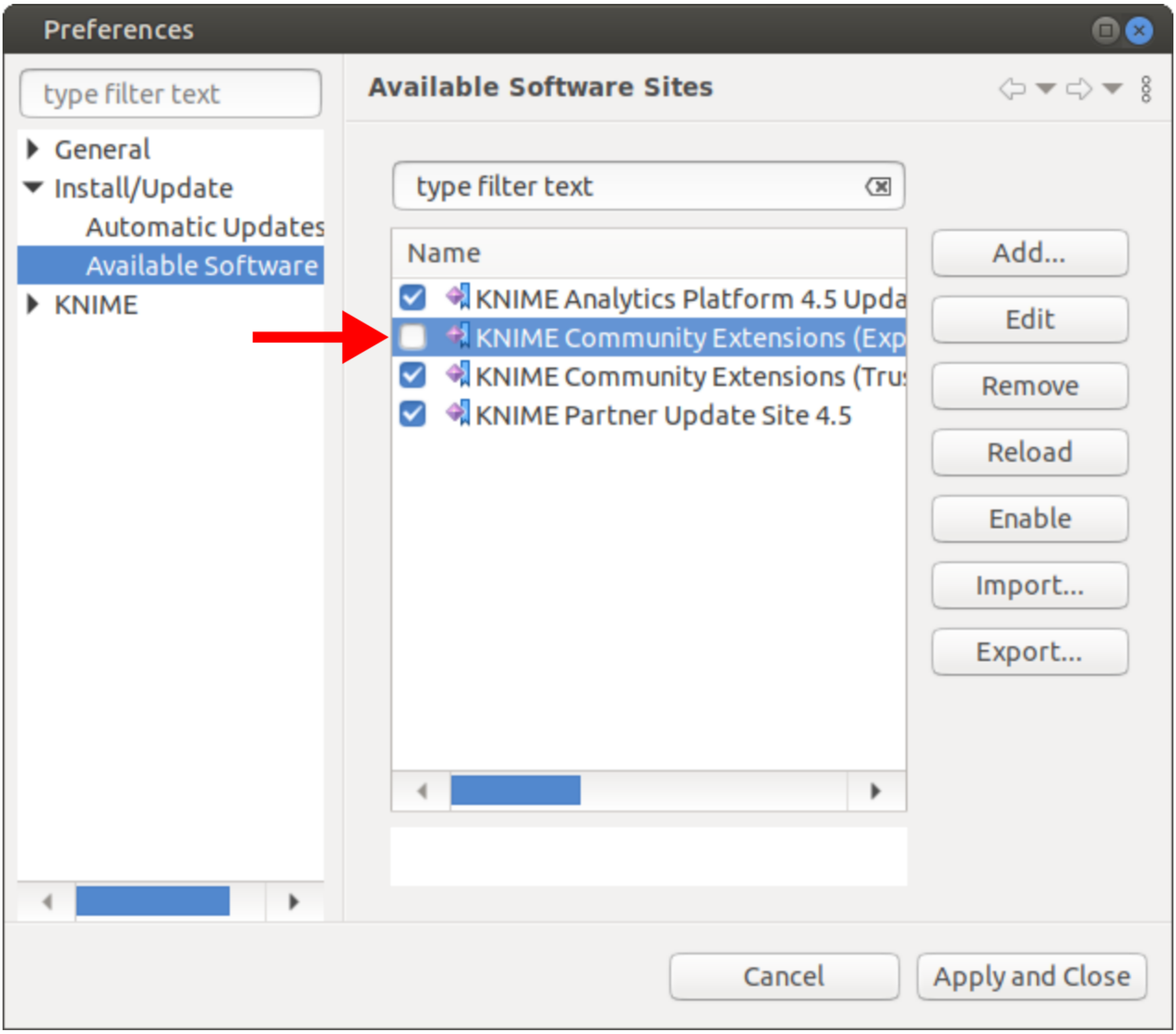

Back in the "Available Software" window select `--All Available Sites--` in the `Work with:` pull down menu.

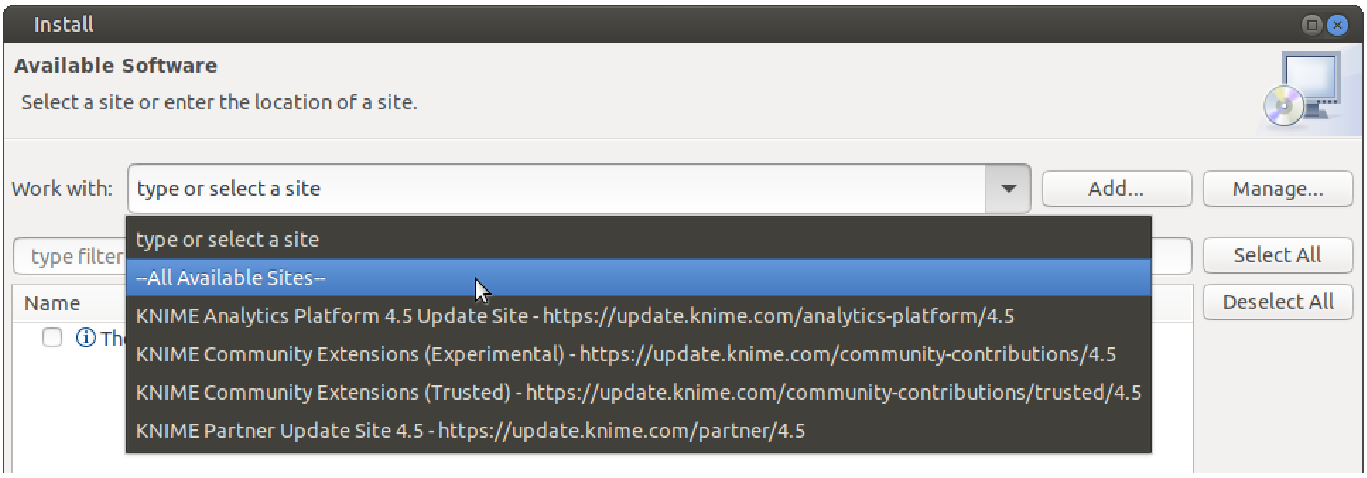

Next search for `imagej` in the search bar and select the `KNIME Image Processing - ImageJ Integration (Beta)` and install the software package by clicking **Next** and **Finish** after accepting the software licenses. You may be prompted again to restart KNIME.

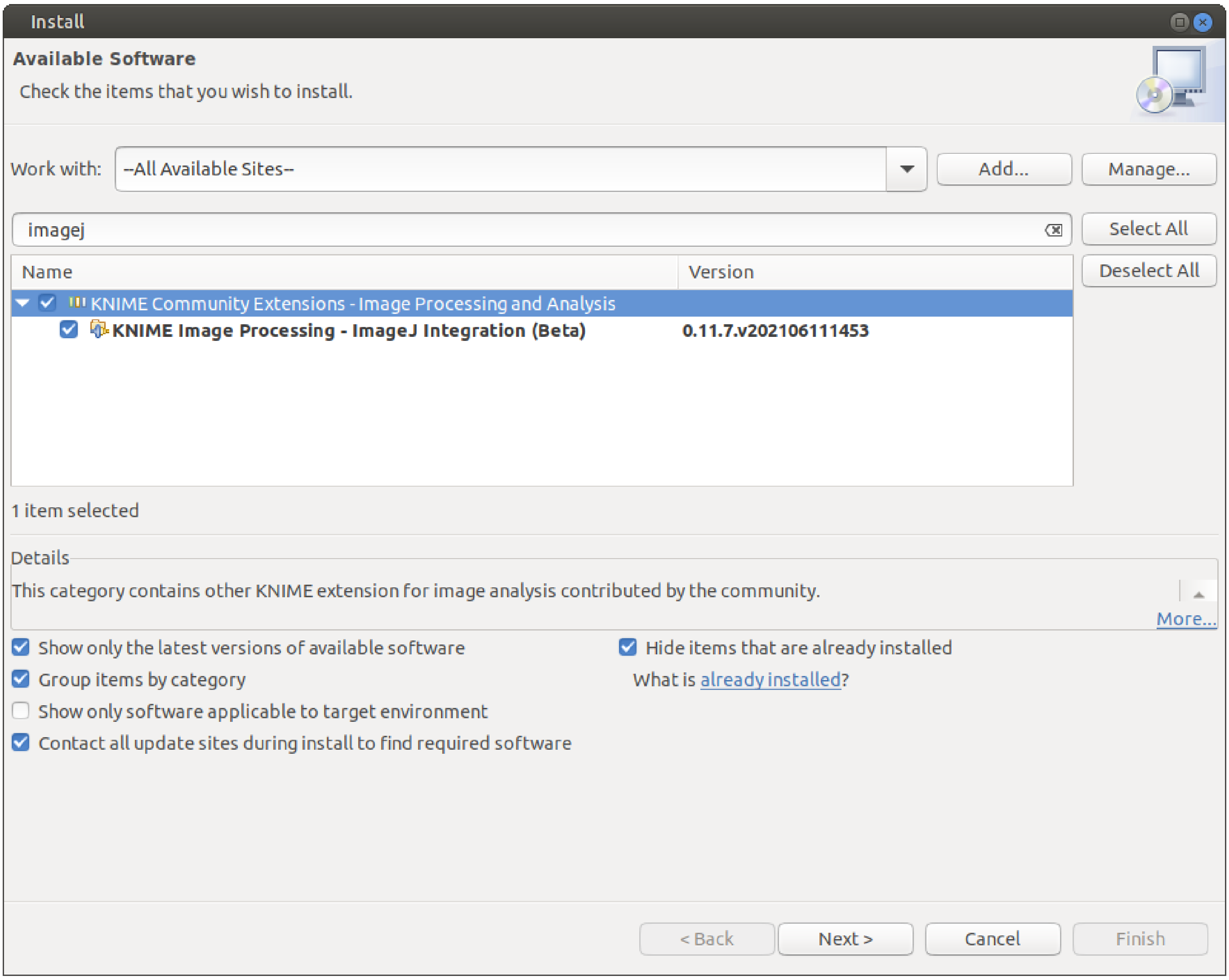

4. Load data into the workflow

To load data into the workflow, right click the first node `Initialize workflow` in the "Folder selection" box and click **Configure...**.

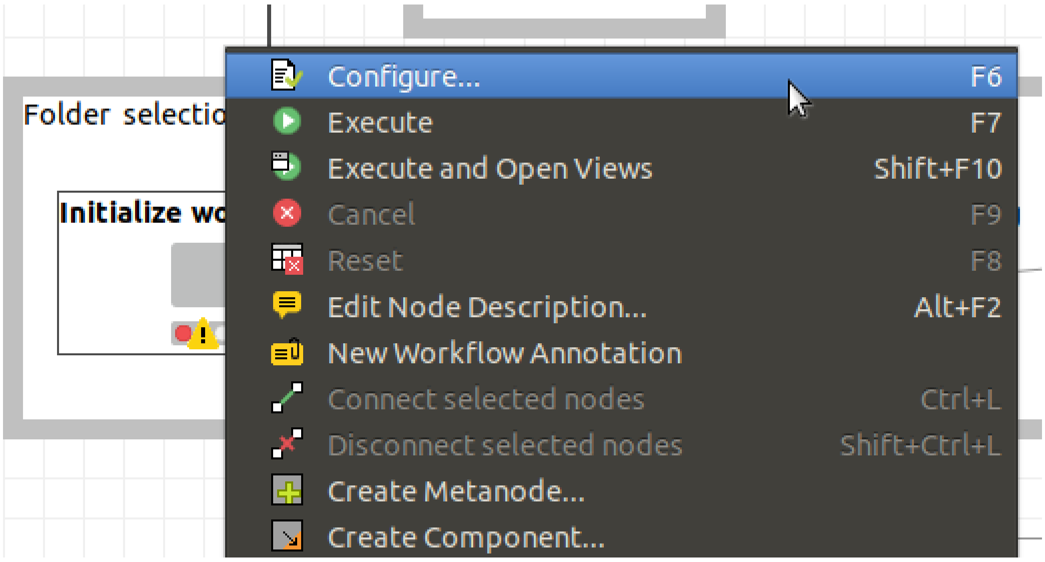

Put the imaging data file into its own **folder**, input the path to the **folder**, then click **OK**.

```
Note: The data file needs to be in its own folder. The NR-SAT workflow will
fail to run if you select the image file itself.
```

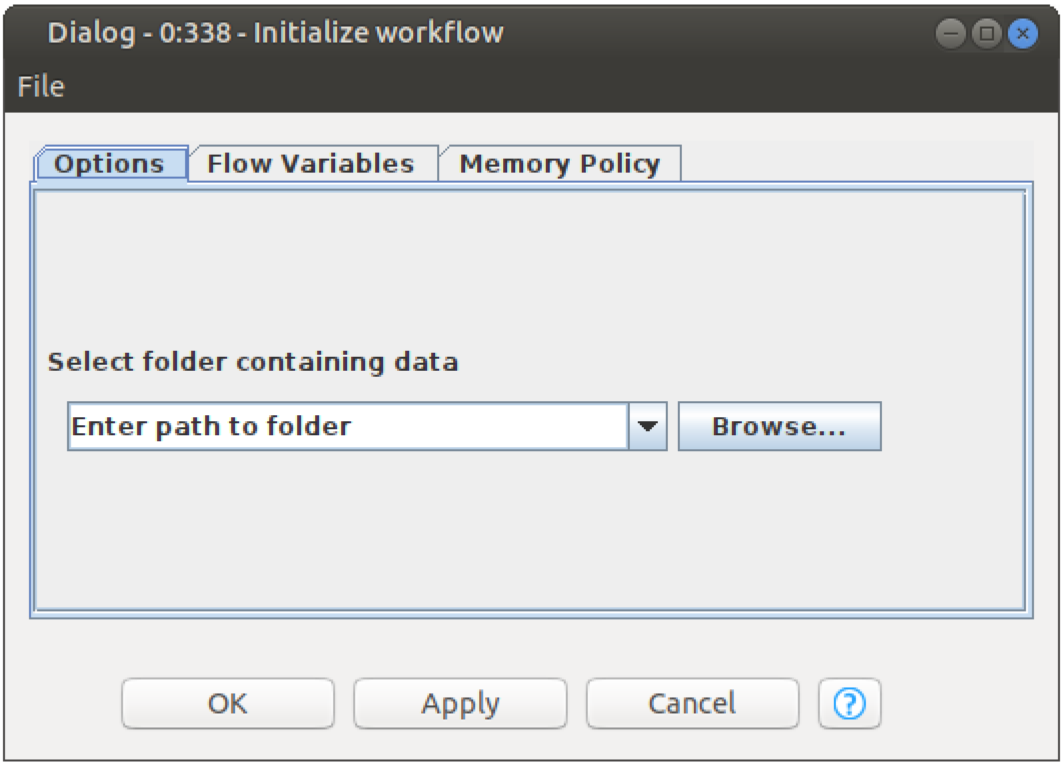

The workflow is now ready to run.

5. Running the workflow

To run workflows click either the single green play button (runs only the selected node and the nodes before the selected node) or the double green play button (runs all nodes in the workflow).

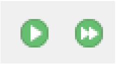

Once your workflow has completed, each node should have a green status. If an error has occurred, look for red nodes with an error icon.

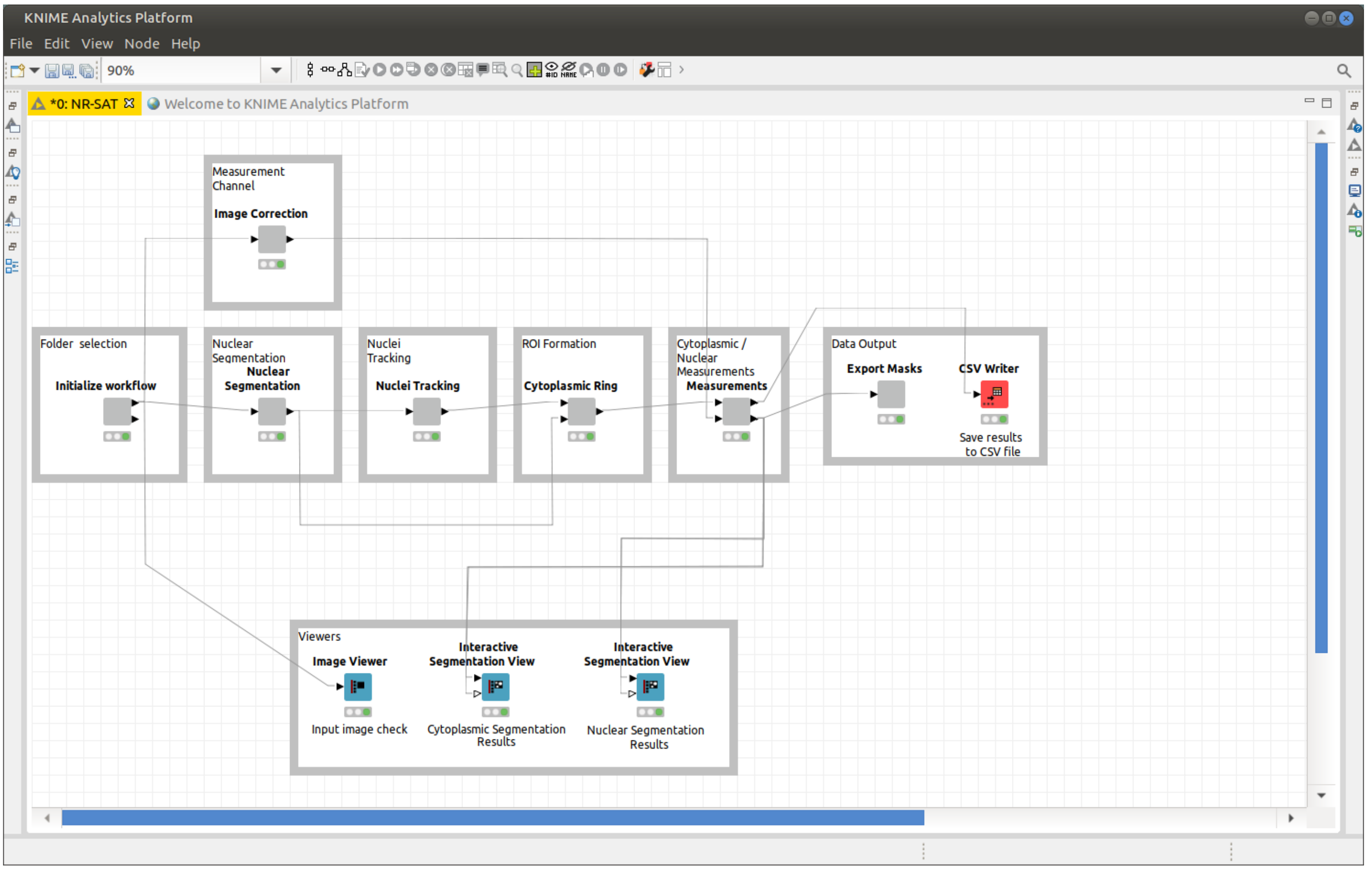

If you are unable to get any output data, check the last two output nodes. If they are red they need to be configured with the correct output directory.

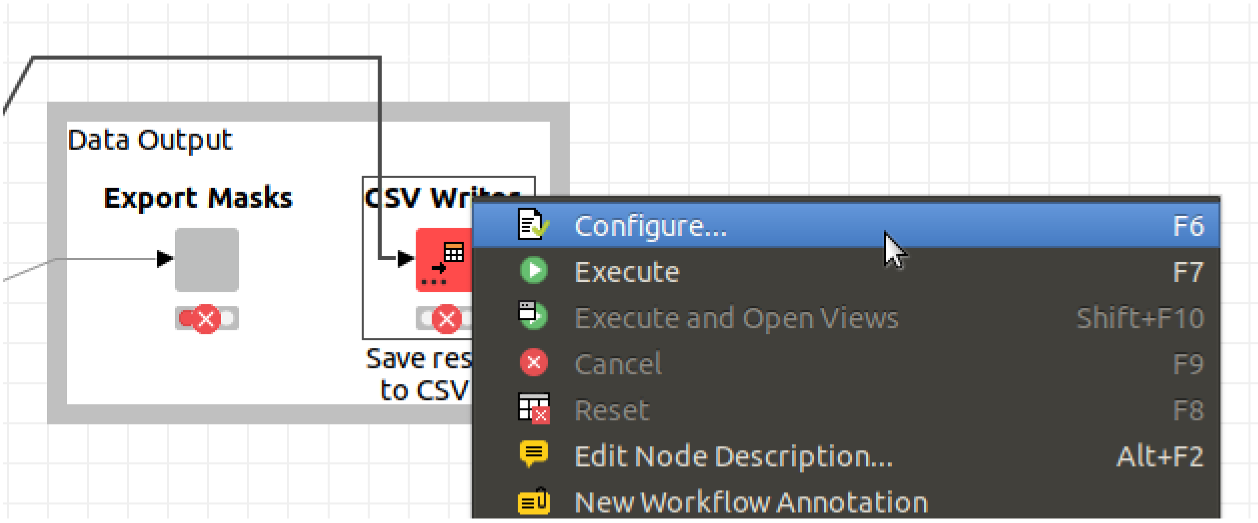

5. Viewing the workflow progress

With most nodes you can view the current state of the data by right clicking the node and selecting **View: Image Viewer**. This will open up a window with the image data and its current processing state (*e.g.* a binary image). You can also utilize the viewers at the bottom of the workflow to check the input data (before any processing is done), the cytoplasmic segmentation and nuclear segmentation.

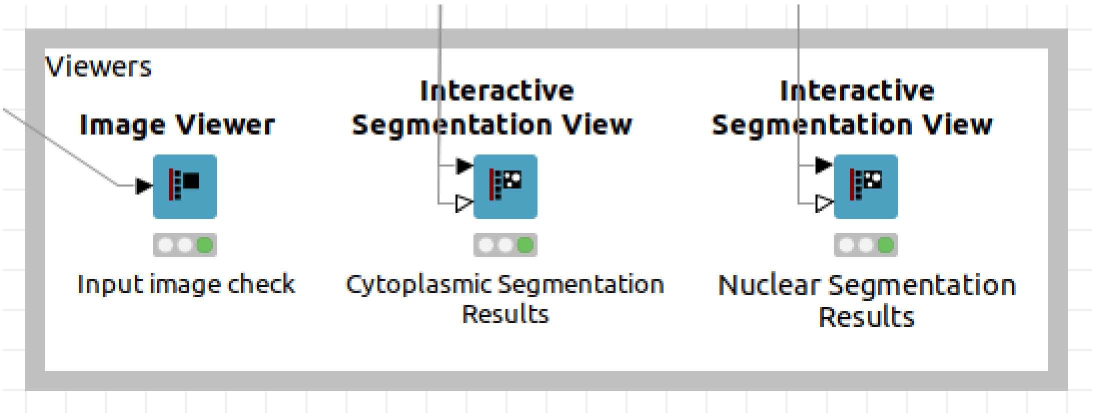

Right click one of the viewers and select **View: Interactive Segmentation View**.

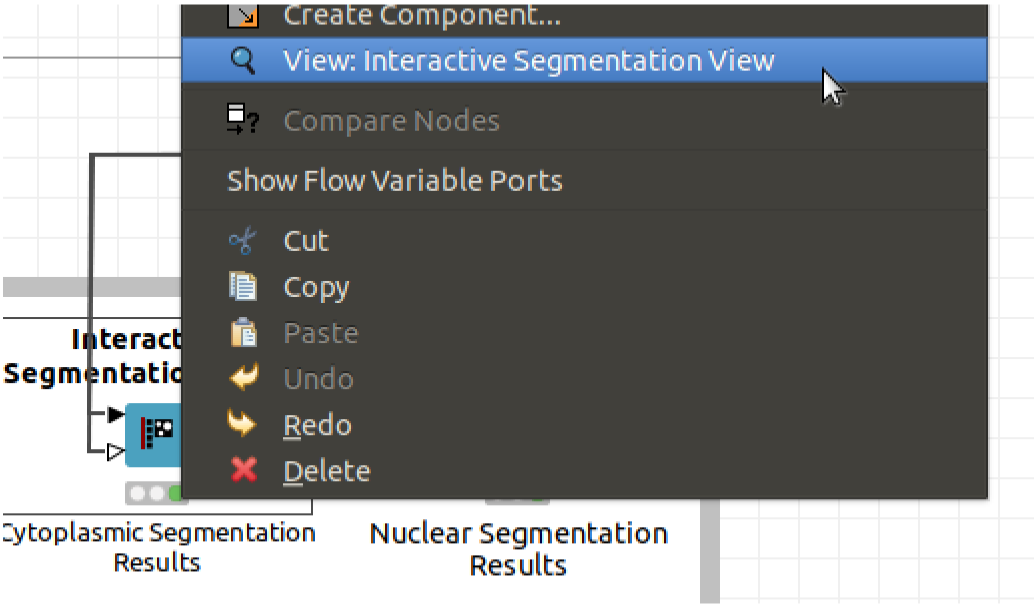

In the interactive segmentation view, double click on either the `Image` or `Labeling` category to open the image inspection window.

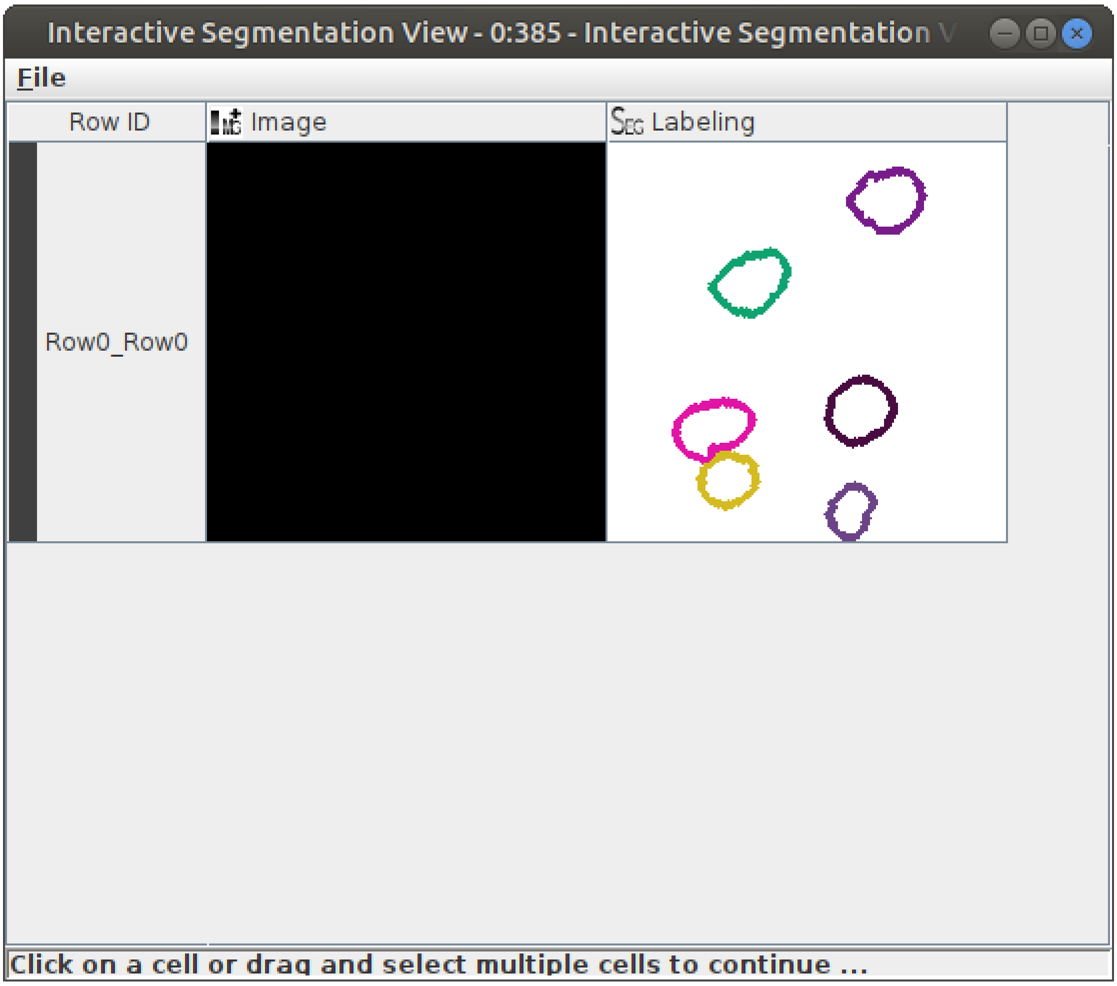
Interactive Segmentation View - 0:385 - Interactive Segmentation V
File
Row ID
Image
Labeling
Row0_Row0
Click on a cell or drag and select multiple cells to continue ...

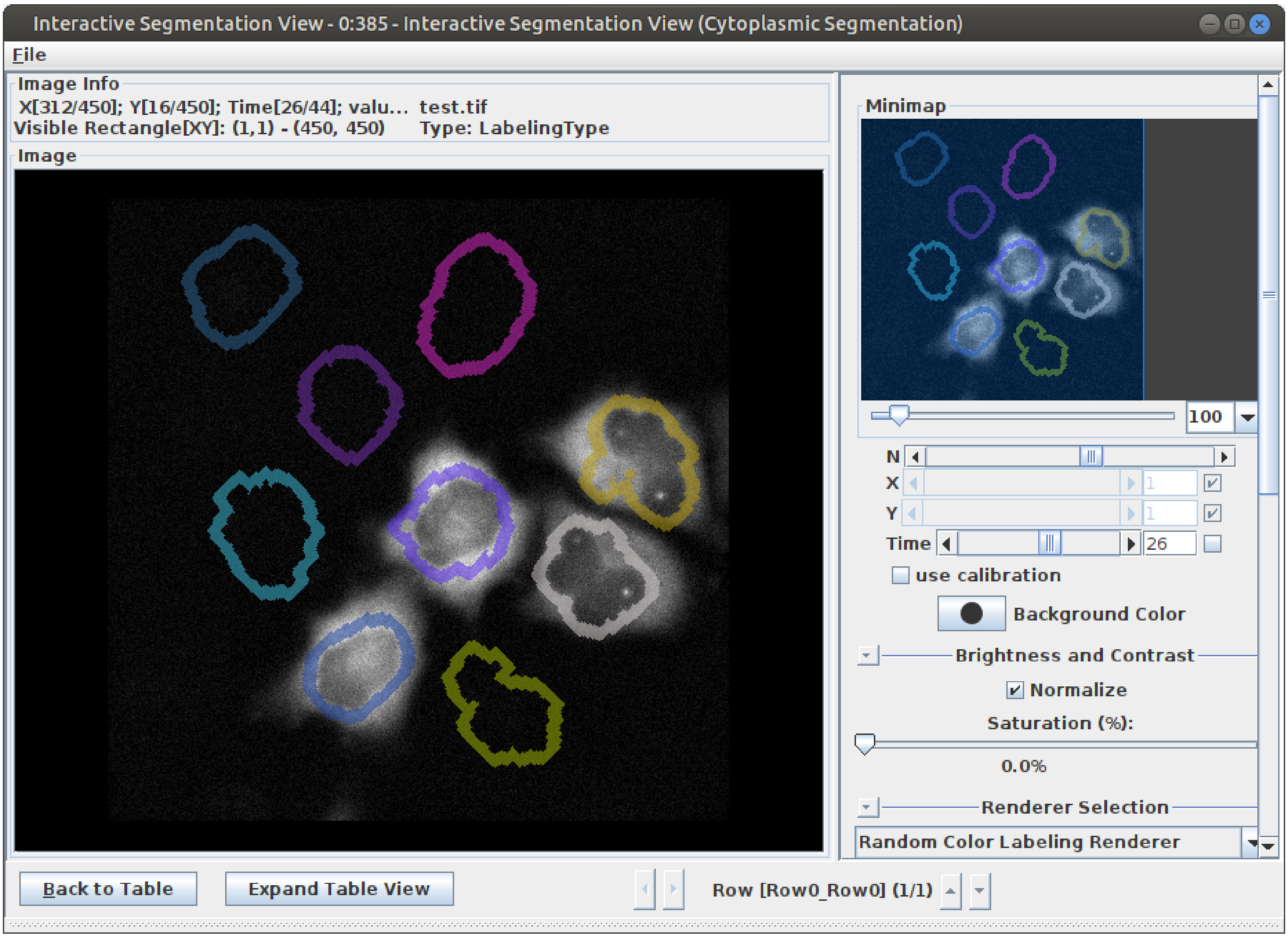
Interactive Segmentation View - 0:385 - Interactive Segmentation View (Cytoplasmic Segmentation)
File
Image Info
X[312/450]; Y[16/450]; Time[26/44]; valu... test.tif
Visible Rectangle[XY]: (1,1) - (450, 450) Type: LabelingType
Image
Minimap
100
N
X
Y
Time
26
use calibration
Background Color
Brightness and Contrast
Normalize
Saturation (%):
0.0%
Renderer Selection
Random Color Labeling Renderer
Back to Table
Expand Table View
Row [Row0_Row0] (1/1)
