# Supplementary figures and images for: HIV RGB: Automated Single-Cell Analysis of HIV-1 Rev-Dependent RNA Nuclear Export and Translation Using Image Processing in KNIME
Source: Viruses. 2022 Apr 26;14(5):903. doi: 10.3390/v14050903 (PMC9145009; doi:10.3390/v14050903)

# Rev-mCherry Trafficking

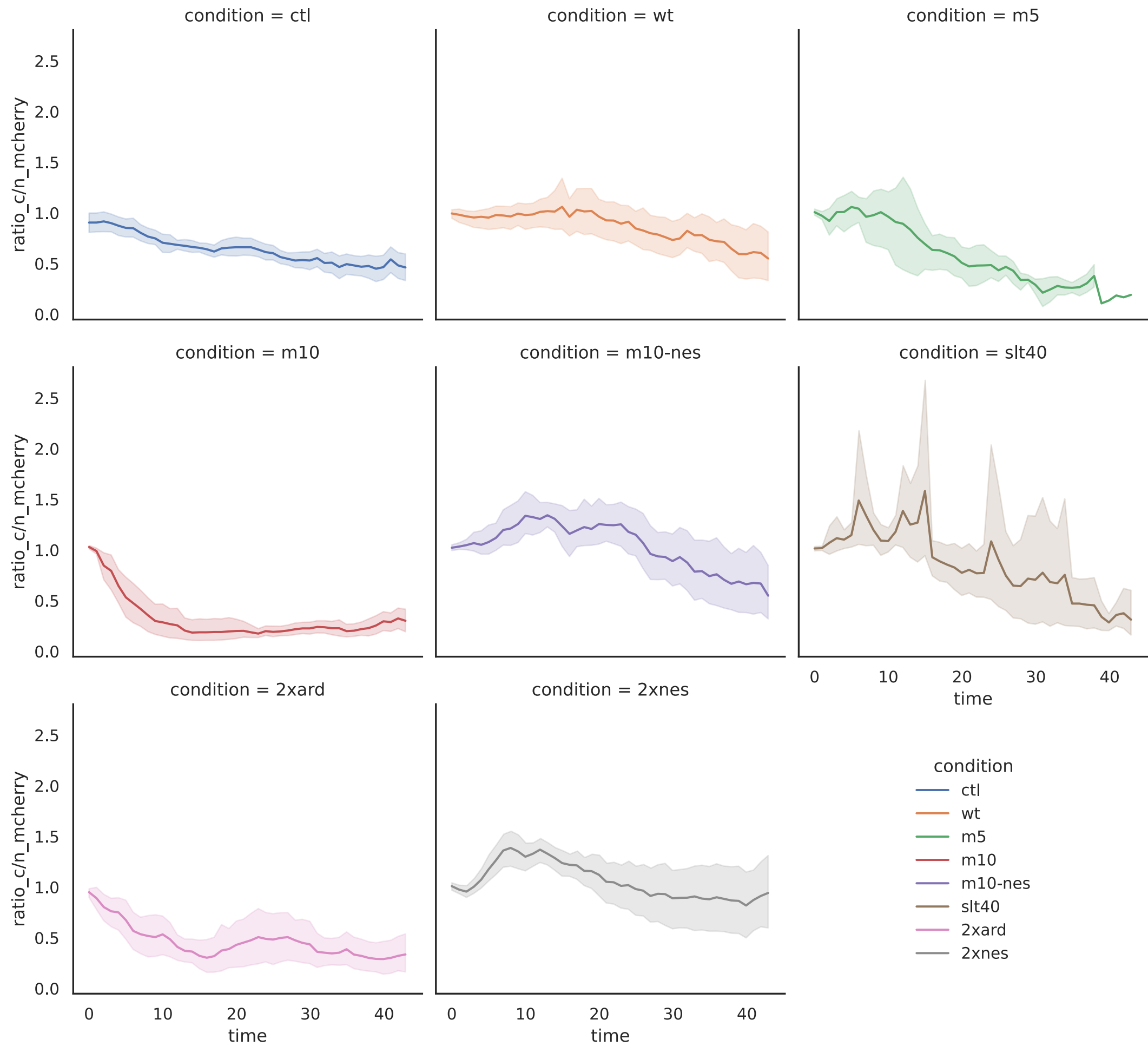

US vRNA (MS2-YFP)

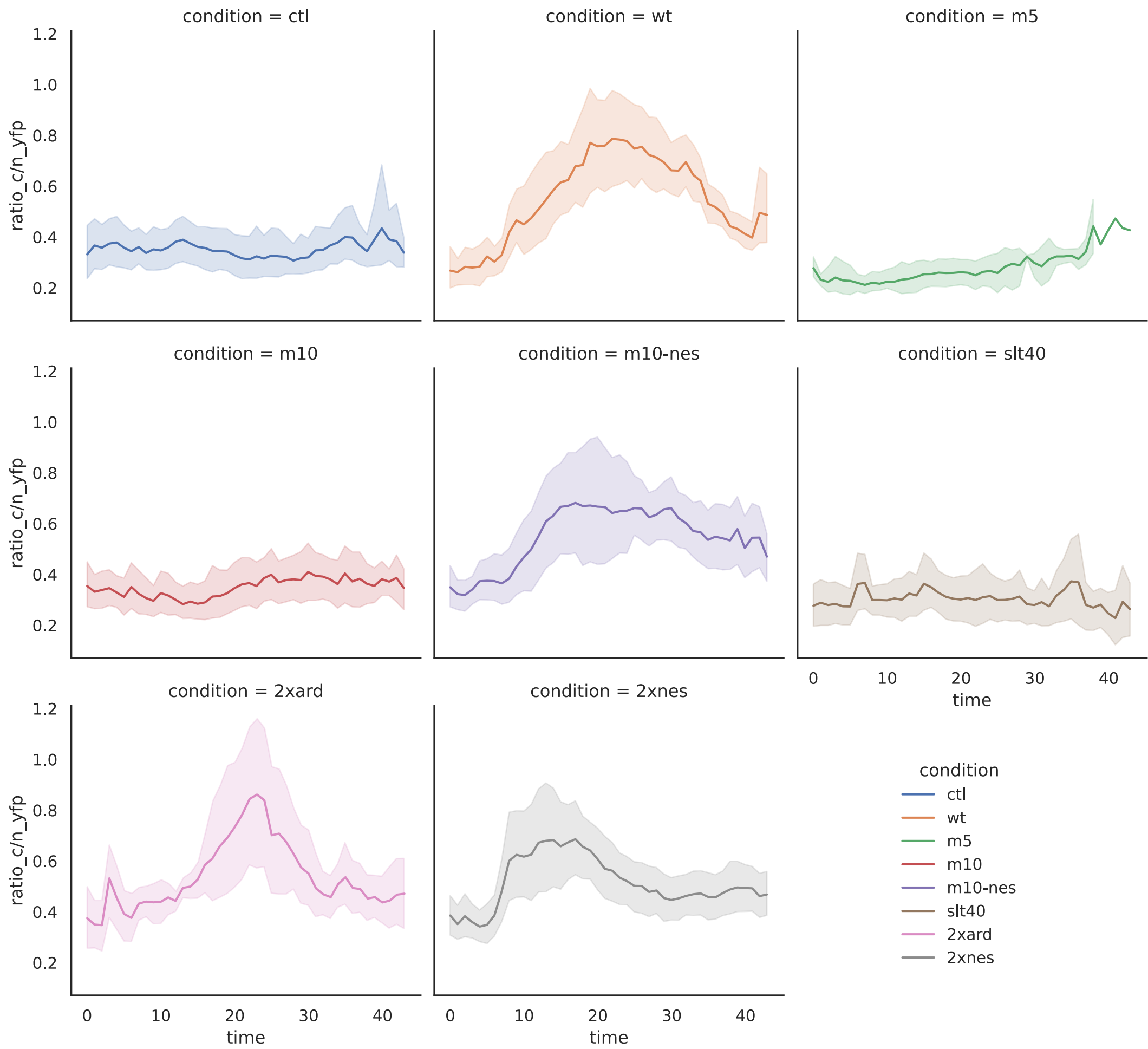

# US-vRNA Translation (Gag-CFP)

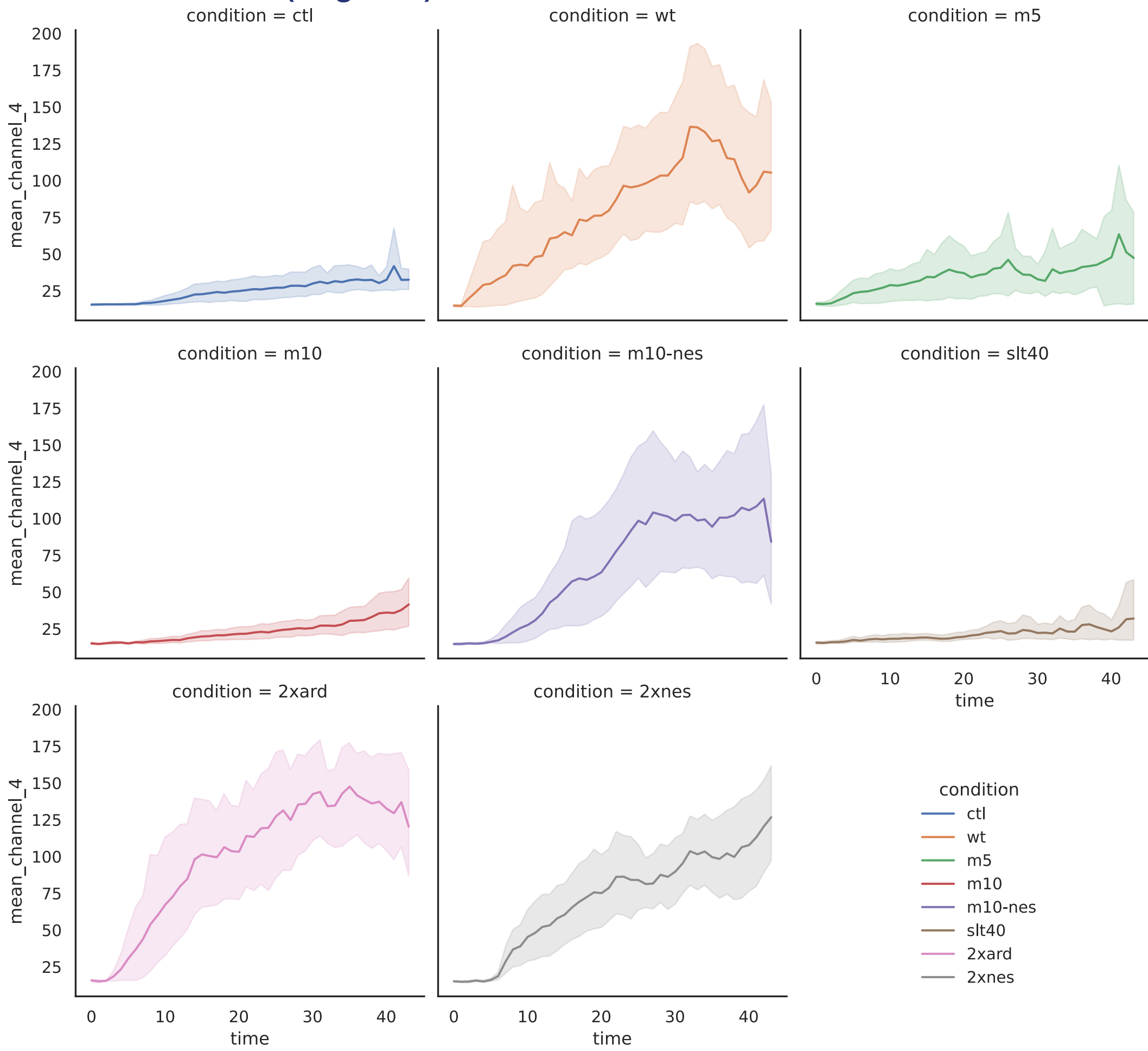

Supplement: Supplementary file 1 [file viruses-14-00903-s001.zip › Supplementary Data File S4.pdf]
